# A national harmonised data collection network for neurodevelopmental disorders: A transdiagnostic assessment protocol for neurodevelopment, mental health, functioning and well‐being

**DOI:** 10.1002/jcv2.12048

**Published:** 2021-11-26

**Authors:** Kelsie A. Boulton, David Coghill, Natalie Silove, Elizabeth Pellicano, Andrew J. O. Whitehouse, Mark A. Bellgrove, Nicole J. Rinehart, Suncica Lah, Marie‐Antoinette Redoblado‐Hodge, Nadia Badawi, Helen Heussler, Nicole Rogerson, Joshua Burns, Michelle A. Farrar, Ralph Nanan, Iona Novak, Micah B. Goldwater, Natalie Munro, Leanne Togher, Natasha Nassar, Phillipa Quinn, Christel M. Middeldorp, Adam J. Guastella

**Affiliations:** ^1^ Autism Clinic for Translational Research Brain and Mind Centre Children's Hospital Westmead Clinical School Faculty of Medicine and Health University of Sydney Camperdown New South Wales Australia; ^2^ Child Neurodevelopment and Mental Health Team Brain and Mind Centre Children's Hospital Westmead Clinical School Faculty of Medicine and Health University of Sydney Camperdown New South Wales Australia; ^3^ Departments of Paediatrics and Psychiatry Faculty of Medicine, Dentistry and Health Sciences University of Melbourne Melbourne Victoria Australia; ^4^ Murdoch Children's Research Institute Melbourne Victoria Australia; ^5^ Child Development Unit The Children's Hospital at Westmead Sydney Children's Hospital Network Westmead New South Wales Australia; ^6^ Macquarie School of Education Macquarie University Sydney New South Wales Australia; ^7^ Telethon Kids Institute University of Western Australia Nedlands Western Australia Australia; ^8^ Turner Institute for Brain and Mental Health School of Psychological Sciences Monash University Clayton Victoria Australia; ^9^ School of Educational Psychology and Counselling Faculty of Education Monash University Clayton Victoria Australia; ^10^ School of Psychology University of Sydney Sydney New South Wales Australia; ^11^ Cerebral Palsy Alliance Research Institute Discipline of Child and Adolescent Health The University of Sydney Sydney New South Wales Australia; ^12^ Grace Centre for Newborn Intensive Care The Children's Hospital at Westmead Sydney New South Wales Australia; ^13^ Child Health Research Centre The University of Queensland Brisbane Queensland Australia; ^14^ Child Development Program Children's Health Queensland Brisbane Queensland Australia; ^15^ Neurodevelopment Australia Sydney New South Wales Australia; ^16^ Sydney School of Health Sciences Faculty of Medicine and Health University of Sydney Sydney New South Wales Australia; ^17^ Discipline of Paediatrics School of Women's and Children's Health UNSW Medicine University of New South Wales Sydney New South Wales Australia; ^18^ Department of Neurology Sydney Children's Hospital Randwick New South Wales Australia; ^19^ Sydney Medical School—Nepean Discipline of Paediatrics University of Sydney Sydney New South Wales Australia; ^20^ Charles Perkins Centre—Nepean The University of Sydney Sydney New South Wales Australia; ^21^ Cerebral Palsy Alliance Research Institute Discipline of Child and Adolescent Health The University of Sydney Sydney New South Wales Australia; ^22^ University of Sydney Camperdown New South Wales Australia; ^23^ Faculty of Medicine and Health University of Sydney Sydney New South Wales Australia; ^24^ Department of Speech Pathology Sydney School of Health Sciences Faculty of Medicine and Health The University of Sydney Sydney New South Wales Australia; ^25^ Child Population and Translational Health Research Children's Hospital at Westmead Clinical School Faculty of Medicine and Health The University of Sydney Sydney New South Wales Australia; ^26^ Child and Youth Mental Health Service Children's Health Queensland Hospital and Health Service Brisbane Queensland Australia

**Keywords:** clinical practice, data sharing, harmonisation, mental health, neurodevelopmental disorders, transdiagnostic

## Abstract

**Background:**

Children with neurodevelopmental disorders share common phenotypes, support needs and comorbidities. Such overlap suggests the value of transdiagnostic assessment pathways that contribute to knowledge about research and clinical needs of these children and their families. Despite this, large transdiagnostic data collection networks for neurodevelopmental disorders are not well developed. This paper describes the development of a nationally supported transdiagnostic clinical and research assessment protocol across Australia. The vision is to establish a harmonised network for data collection and collaboration that promotes transdiagnostic clinical practice and research.

**Methods:**

Clinicians, researchers and community groups across Australia were consulted using surveys and national summits to identify assessment instruments and unmet needs. A national research committee was formed and, using a consensus approach, selected assessment instruments according to pre‐determined criteria to form a harmonised transdiagnostic assessment protocol.

**Results:**

Identified assessment instruments were clustered into domains of transdiagnostic assessment needs, which included child functioning/quality of life, child mental health, caregiver mental health, and family background information. From this, the research committee identified a core set of nine measures and an extended set of 14 measures that capture these domains with potential for further modifications as recommended by clinicians, researchers and community members.

**Conclusion:**

The protocol proposed here was established through a strong partnership between clinicians, researchers and the community. It will enable (i) consensus driven transdiagnostic clinical assessments for children with neurodevelopmental disorders, and (ii) research studies that will inform large transdiagnostic datasets across neurodevelopmental disorders and that can be used to inform research and policy beyond narrow diagnostic groups. The long‐term vision is to use this framework to facilitate collaboration across clinics to enable large‐scale data collection and research. Ultimately, the transdiagnostic assessment data can be used to inform practice and improve the lives of children with neurodevelopmental disorders and their families.

**Key points**

**What's known?**

- Children with neurodevelopmental disorders and their families share many common needs. National and international harmonised data collection networks across neurodevelopmental disorders are not well‐developed.

**What's new?**

- We present a consensus driven transdiagnostic assessment protocol with researchers, clinicians and community groups and across many specialist clinics.

**What's relevant?**

- This protocol is proposed to be used nationally and internationally to establish one of the first large scale transdiagnostic databases.
- This protocol enables forward and back translation between research and practice and across child neurodevelopment and functioning. The data arising from this protocol can be used to inform policy and practice, ultimately improving the lives of children with neurodevelopmental disorders and their families.

## INTRODUCTION

It is estimated that one in 10 children meet diagnostic criteria for at least one neurodevelopmental disorder (Arabiat et al., 2018; McGuire et al., 2019). These disorders are often lifelong and impact cognitive, social and emotional development from the first years of life onwards (American Psychiatric Association, 2013). The most frequent referrals for neurodevelopmental assessment include global developmental delay, autism spectrum disorder (autism), attention‐deficit hyperactivity disorder (ADHD) and specific learning difficulties (e.g., dyslexia), but may also include cerebral palsy, Tourette's syndrome, single gene disorders (e.g., Fragile X Syndrome), and language disorders (Boyle et al., 2011; Hiscock et al., 2011; Kayrouz et al., 2014; Lo et al., 2017).

It is well established that children with neurodevelopmental disorders who present to health services will commonly receive a single primary diagnosis that guides future treatment (Hiscock et al., 2017; McDowell, 2018). However, diagnostic overshadowing, where symptoms are attributed to an identified condition rather than being considered as indicators of comorbid conditions, is common and occurs irrespective of the complexity with which an individual presents (Hendriksen et al., 2015; Kerns et al., 2015). As a result, clinical practice has been historically narrow in focus and fragmented by professional and diagnostic silos in which clinicians prioritise the needs of a specific neurodevelopmental disorder but fail to address those concerns and difficulties that fall outside their familiarity or expertise. This often means that caregivers will be required to seek treatment for their child in different places to fully meet their needs. This can lead to increased difficulties accessing and navigating services, unnecessary delays in obtaining supports or a complete absence of a response to these needs (Thapar et al., 2017). These specialist silos can be perpetuated by diagnostic criteria. For instance, until the release of the DSM‐5, a child could not be diagnosed with both autism and ADHD despite the high rates of overlap seen both clinically and in research (Gargaro et al., 2011).

A similar diagnosis‐specific pattern is present in research, where there are many networks focusing on single neurodevelopmental disorders but far fewer with a transdiagnostic focus. The EU‐AIMS (Murphy & Spooren, 2012), SPARK (Feliciano et al., 2018) and Australian Autism Biobank (Alvares et al., 2018) networks in Europe, the United States and Australia, respectively, have established some of the largest networks for the collection of genomic, cognitive and behavioural data to inform diagnosis and clinical interventions for autism. Likewise, the Australian Cerebral Palsy Register has been established to facilitate large‐scale data collection to inform on aetiology, outcomes and health service planning in cerebral palsy (Australian Cerebral Palsy Register, 2016). This work has resulted in many impactful research outcomes for children and families. For example, cerebral palsy research networks have substantially improved the detection, intervention, rate and severity of cerebral palsy diagnosis in young children over the last 20 years (King et al., 2020; Novak et al., 2017). Similarly, autism research and clinical networks have substantially advanced our knowledge about early intervention and support for social development, as well as current intervention patterns for autistic children and adolescents (Hazlett et al., 2017; Loth et al., 2017; Monz et al., 2019). Although these networks do consider comorbidity in their measurements, the fact that they focus on one particular disorder precludes transdiagnostic research into predictors or treatment of symptoms that may be prevalent across disorders.

It is abundantly clear that transdiagnostic assessment and diagnosis pathways are needed in research and clinical practice. Adopting a transdiagnostic approach requires assessment of symptoms, functioning and social factors that may be shared across multiple neurodevelopmental disorders (Finlay‐Jones et al., 2019; Thapar et al., 2017). This dimensional approach enables better understanding of needs beyond a single diagnosis and aligns with the National Institute of Mental Health Research Domain Criteria (RDoC) framework (Casey et al., 2014). For example, it is well established that comorbidity in presentation is the rule rather than the exception in neurodevelopmental disorders. Across clinical and population samples, it has been estimated that at least 80% of children with one neurodevelopmental disorder experience multiple, co‐occurring neurodevelopmental and psychiatric disorders, as well as far higher rates of other medical comorbidities (e.g., epilepsy, sleep problems and cardiovascular disorders) (Antshel & Russo, 2019; Hiscock et al., 2017; King, 2016; Pahlman et al., 2020; Rommelse et al., 2010). There is also growing evidence that children with sub‐threshold presentations have much higher rates of mental health problems and impairments than the general population, which are associated with negative educational outcomes and poorer functional outcomes in adulthood (Copeland et al., 2015; Zendarski et al., 2020). Furthermore, there is a high prevalence of mental health problems in caregivers of children with neurodevelopmental disorders (Bromley et al., 2004; Masefield et al., 2020).

Basic science also supports the potential of using transdiagnostic frameworks. Aside from single‐gene disorders, neurodevelopmental disorders are largely considered polygenic, sharing common genetic variations that increase risk, and may be related to symptoms and levels of functioning (Niemi et al., 2018; Sengupta et al., 2018). Similarly, environmental variables linked to neurodevelopmental risk, such as prematurity, low birth weight, increased parental age, compromised prenatal environment and lower socioeconomic status have been associated with multiple neurodevelopmental disorders (Carlsson et al., 2020; Han et al., 2021; Sciberras et al., 2017).

### Development of a harmonised, transdiagnostic data collection network for neurodevelopmental disorders

There is an emerging consensus that research would benefit more through harmonisation and consolidation of data across neurodevelopmental disorders (Casey et al., 2014; Thapar et al., 2017). Likewise, the importance of harmonised data collection as part of standard clinical practice is beginning to be acknowledged (Krause et al., 2021). There is growing recognition that children with neurodevelopmental disorders share common needs that have significant impacts on individual and family functioning and developmental outcomes. The importance of this in policy has been recently highlighted by funding bodies. For example, Australia's National Disability Insurance Agency (NDIA) criteria for funding has moved towards a diagnostically agnostic model, with a diagnosis‐neutral suite of assessment tools to identify support needs (NDIA, 2020). This diagnosis‐agnostic approach aligns with the International Classification of Functioning, Disability and Health (ICF) framework which also supports universally accepted frameworks to describe functioning across different conditions and groups (WHO, 2001).

Despite this growing awareness, internationally, we are not aware of any agreed data collection protocols and/or platforms that are integrated with clinical services and facilitate the collection of standardised, transdiagnostic information from the broad group of children with neurodevelopmental disorders and their families. Such a platform would have the potential to facilitate stronger and more coherent links between research and clinical services for children with neurodevelopmental disorders. Identification of transdiagnostic needs will provide clinicians with knowledge of concerns and needs that extend beyond the primary diagnoses. Identification of these needs will provide clinically actionable options that may enable a more integrative approach to care and improve patient outcomes. Recent findings indicate that there is strong enthusiasm and a preference for standardised electronic data collection amongst families of children with neurodevelopmental disorders (Patel et al., 2021). As such, an electronic data collection platform may have a particularly high chance of successful uptake in this cohort. In keeping with the move toward large, unified datasets that can inform both research and clinical practice, such as those used in youth mental health (Lavoie et al., 2020), the aim of this paper was to propose an assessment protocol for harmonised data collection across research centres, hospital‐based and community clinics in the field of child neurodevelopment. It was also to establish this protocol in collaboration with a national consortium of researchers, clinicians, and community representatives with extensive experience in the field of child neurodevelopment from across Australia.

## METHODS

A consortium of researchers, clinicians, people with lived experience and community members working in the field of child neurodevelopment in Australia, was established under the banner of ‘Neurodevelopment Australia’, to facilitate the development of large‐scale data collection and linkage, collaborative opportunities and translational, multi‐site research. Members were invited based on their expertise, experience, and background in the field of child neurodevelopment and neurodevelopmental disorders. A key goal of the Neurodevelopment Australia consortium was to develop a protocol for standardised data collection across public hospital and community clinics, community groups and research centres who provide services for children with neurodevelopmental disorders and their families. Using a streamlined protocol would enable researchers to address questions about specific disorders, as well as transdiagnostic questions that reach across disorders. Additional consultation was also sought from the Child Neurodevelopment and Mental Health Team from the University of Sydney. The COS‐STAR (Core Outcome Set‐Standards for Reporting) guidelines were followed when developing the transdiagnostic assessment protocol (see Table S1).

### Engagement with researchers and clinicians

Twenty‐six individuals including research academics, psychiatrists, psychologists, paediatric neurologists, neonatologists, paediatricians, epidemiologists, speech pathologists and not‐for‐profit disability organisation employees took part. They reported on the questionnaires and tools they currently use and those that they felt should be used to harmonise data collection across neurodevelopmental disorders. These individuals came from 23 speciality clinics based at universities or children's hospitals. In total, 14 institutions and hospitals across Australia were represented.^1^ Combined, these institutions and hospitals see over 6000 children with neurodevelopmental disorders per year.

Experts were asked to provide information about: (i) the clinical populations they serve (e.g., autism, ADHD); (ii) the estimated proportion of comorbidities seen in presenting patients; (iii) the assessment tools and measures currently being used; (iv) the method by which assessments were being conducted (paper/pencil or electronically); (v) assessment tools and measures that would have transdiagnostic utility across neurodevelopmental clinics/institutes; and (vi) domains that were not currently being measured, but which respondents thought should be measured across neurodevelopmental disorders.

### Engagement with the broader neurodevelopmental community

In addition to consulting experts in the field of neurodevelopmental disorders, members of the neurodevelopmental community were consulted, to better understand their concerns and needs and to inform how these needs can be addressed using a transdiagnostic approach. In total, 11 community organisations across Australia were represented^2^. Community members comprised youth and adults with lived experience of a neurodevelopmental disorder, parent advocates, and representatives of these organisations.

Community members were invited to a half‐day community summit. Attendees at the community summit engaged in open discussion and were asked to provide feedback on: (i) their experiences with service providers; (ii) what they felt was missing from their interactions with hospital and diagnostic services; (iii) the needs and vulnerabilities of the neurodevelopmental community; and (iv) the potential value of transdiagnostic supports across their communities.

### Development of harmonised assessment protocol

Following input from researchers, clinicians, and the community, a research committee (comprised of authors Nadia Badawi, Mark A. Bellgrove, David Coghill, Michelle A. Farrar, Adam J. Guastella, Helen Heussler, Christel M. Middeldorp, Elizabeth Pellicano, Nicole J. Rinehart and Andrew J. O. Whitehouse) was formed to reach a consensus on the domains that should be measured using a transdiagnostic assessment protocol, and the specific assessment tools and measures that would comprise each domain. Research committee members were selected from within Neurodevelopment Australia based on their research and clinical expertise in the field of child neurodevelopmental disorders. The research committee met face‐to‐face, via teleconference, and through email over a period of nine months to reach consensus on domains and specific measures. The technique used to reach consensus was a variant of the Nominal Group Technique. Our approach was similar to that used by Lavoie et al. (2020) when developing a core data set for youth mental health. Research committee members were asked to review the information collected from researchers, clinicians and the broader neurodevelopmental community. Following this, they were asked to answer the following questions: ‘What domains should be assessed transdiagnostically in child neurodevelopment’, and ‘What measures should be used to capture these domains?’. Research committee members were asked to use the information collected and combined this with the wider literature when discussing and making recommendations. All domains and measures were considered by research committee members, with the aim being the creation of an assessment protocol that was appropriate for children aged 0–18 years, irrespective of the specific diagnosis or problems the child was presenting with.

Several criteria were used to select measures for inclusion in the assessment protocol. First, measures needed to be applicable across neurodevelopmental disorders and, where possible, relevant to children and adolescents aged from 0 to 18 years. Second, given the patient‐facing time restrictions of many developmental clinics, there was a preference for caregiver‐completed measures that were questionnaire‐based, with the ability for completion prior to assessment (e.g., via electronic data capture). Third, efforts were made to select measures with sound psychometric properties and where possible, those that have been validated in children and adolescents with neurodevelopmental disorders. Finally, there was a preference for measures that are freely available and in the public domain wherever possible, to ensure ease of access.

To keep the core transdiagnostic assessment protocol as brief as possible, and thereby accessible and attractive to a wider range of hospital and community‐based clinics, research committee members were advised to present a protocol that would take no longer than 45 min to complete. Additional domains and measures considered to be transdiagnostically relevant, but that are substantially longer and/or not accessible in the public domain, were incorporated into an extended transdiagnostic assessment protocol. The rationale for an extended protocol was to allow collection of additional information in clinics with capacity and resources to do so, thereby enabling harmonisation of these measures as well. As the requirements for assessment are quite different from the requirements for monitoring change, a conscious decision was made not to use ability to track change as a selection criterion. A separate outcome set will be developed to measure outcomes and monitor the changing needs of children and families with neurodevelopmental disorders over time.

## RESULTS

### Feedback from researchers and clinicians – Assessments used in clinics

As shown in Table 1, researchers and clinicians from 23 public clinics provided information about the measures and tools they routinely use to assess presenting children. Clinic specialities included: movement disorders (e.g., Tourette's syndrome); cerebral palsy; early developmental signs (patients are infants admitted to the neonatal intensive care unit who are at risk of developmental problems); developmental assessment (patients are children who have been referred for assessment and evaluation due to suspected developmental delay/intellectual disability); eating disorders; autism; ADHD; neuromuscular disorders and epilepsy. The age range of children across the clinics was 0–18 years (see Table S2, for a breakdown of the age range per speciality). Given the high rates of comorbidities across clinical specialties (e.g., experts indicated that approximately 10% of patients presenting to eating disorder clinics present with comorbid autism), a broad range of specialties were included, to capture the diversity of children seen across settings.

**TABLE 1:** Information collected in surveyed public developmental clinics as standard practice

Clinic speciality	Information collected across clinics					
	Diagnostic interview/medical assessment	Symptom/behaviour screen	Cognitive/developmental assessment	Adaptive functioning	Parental screen	Motor functioning
Movement disorders	Y	Y	–	Y	Y	–
Cerebral palsy	Y	Y	–	–	–	Y
Early developmental signs^a^	Y	–	Y	–	–	Y
Developmental assessment^b^	Y	Y	Y	Y	–	–
Eating disorders	Y	Y	–	–	–	–
Autism	Y	Y	Y	Y	Y	Y
ADHD	Y	Y	Y	Y	Y	–
Neuromuscular disorders	Y	–	–	–	–	–
Epilepsy	Y	–	Y	–	–	–
Total percentage	100%	67%	56%	44%	33%	33%

Abbreviations: ADHD, attention‐deficit hyperactivity disorder; Y, Yes.

^a^ Early developmental signs – Patients are children who were in the neonatal intensive care unit. Patients are seen routinely until 3.5 years of age.

^b^ Developmental assessment – Patients are children who have been referred to an assessment clinic with a suspected developmental delay requiring evaluation.

Although individual assessments were extensive and varied considerably across specialties (see Table S3, for a full list of measures used), it became apparent that each speciality focussed on similar domains as part of their routine assessment. As displayed in Table 1, a diagnostic interview and/or medical assessment was performed across all specialities, while other domains varied as a function of speciality. For example, motor functioning was measured in clinics specialising in cerebral palsy, where some degree of motor difficulties are commonly seen in presenting children, whereas cognitive assessments were more often conducted in clinics specialising in global developmental delay, autism and ADHD, where a report of cognitive functioning is typically required for monitoring purposes.

The same group of researchers and clinicians provided feedback on assessment measures and tools they believed would have transdiagnostic utility across all developmental clinics. Each respondent also provided feedback on the domains that were not currently being measured as part of their standard practice, but which they thought should be measured transdiagnostically across clinics. While the individual measures that were recommended varied across specialties (see Table S4, for a full list of measures recommended by each specialty), there was consistency on the domains recommended for transdiagnostic assessment across specialities. As displayed in Table 2, most specialities recommended that information relating to functioning and quality of life should be captured as part of standard clinical practice. However, fewer than half of these specialities reported that they routinely captured these outcomes, highlighting the gap between the data clinicians believe should be captured, and the data that are currently being captured as part of standard clinical practice.

**TABLE 2:** Domains recommended as standard practice across diagnostic groups by public developmental clinics

	Domains recommended by clinics				
Clinic speciality	Functioning/quality of life	Family background information	Caregiver mental health	Child behaviour/symptoms	Cognitive functioning/child development
Movement disorders	Y	Y	Y	Y	Y
Cerebral palsy	Y	–	–	–	–
Early developmental signs^a^	–	Y	–	–	–
Developmental assessment^b^	Y	Y	Y	Y	Y
Eating disorders	Y	–	–	–	–
Autism	Y	Y	Y	Y	–
ADHD	Y	Y	Y	Y	–
Neuromuscular disorders	Y	Y	Y	–	–
Epilepsy	–	–	–	–	–
Total percentage	78%	67%	56%	44%	22%

Abbreviations: ADHD, attention‐deficit hyperactivity disorder; SES, socioeconomic status; Y, Yes.

^a^ Early developmental signs – Patients are children who were in the neonatal intensive care unit. Patients are seen routinely until 3.5 years of age.

^b^ Developmental assessment – Patients are children who have been referred to an assessment clinic with a suspected developmental delay requiring evaluation.

### Community experiences, concerns and needs

Community members comprised 24 individuals (young people and adults with lived experience, parents, representatives from community organisations), providing representation across a broad range of developmental conditions, including autism, ADHD, Tourette's syndrome, Cerebral Palsy and Down syndrome. At the summit, there was discussion of the assessment measures routinely used by developmental clinics and the measures that clinics reported as having transdiagnostic utility across developmental clinics. Community members raised a range of suggestions and issues for consideration at the summit. Many suggestions extend beyond the scope of this paper and will be expanded upon in future work. Key insights raised included the importance of harmonising research, clinical practice, and the community with a transdiagnostic framework that can address needs that extend beyond diagnostic specialisation. Community members also emphasised the importance of utilising technology to assess needs in the neurodevelopmental space and focusing on functional abilities independent of a specific diagnosis. Following the community summit, community members, clinicians and researchers established key principles for further development in the field of transdiagnostic research and practice. These principles are presented in Table 3.

**TABLE 3:** Community‐recommended principles for further development in transdiagnostic research and practice

Principle	Description
Adopting a transdiagnostic approach	Working in diagnostic and professional silos is not effective. By collaborating and cooperating across disorders, there is potential to learn more about commonalities and differences in disorders, and to have a much larger impact on research and practice
	There is enormous potential for community organisations to learn from each other and for the strengths of one organisation to benefit other groups and organisations by working together. Certain organisations have particular strengths due to the focus of support for that diagnosis, which can be shared transdiagnostically to benefit the broader community (e.g., Tourette's Australia highlighted strengths of practice in running sibling and community camps; Amaze discussed their post‐diagnosis support network)
Identifying and attending to individual needs and developing individualised care plans	The individual needs of a child beyond their specific diagnosis should be considered. Families should be provided with roadmaps to services and support that are tailored to their child's needs. While a specific diagnosis is helpful for families to understand some of the supports their child will require, addressing individual needs will likely improve outcomes for children and families, as well as their overall interactions with service providers
Focus on everyday functioning	Services should focus on a child's level of functioning, rather than just the diagnostic label. The focus should be on what the child's level of functioning is, and what critical supports they require based on that level of functioning
Harness technology	Technological advances should be better utilised in the neurodevelopmental space. Assessing needs, measuring outcomes and providing support to families should be done using technology wherever possible, for example, via electronic data collection

### Development of harmonised assessment protocol – Selection of assessment measures

To reach consensus, members of the research committee first reviewed the measures currently being used across developmental clinics and institutes, and the domains and measures that experts recommended as being of transdiagnostic relevance. From this review, four key domains were identified and agreed upon: child functioning/quality of life; child mental health; caregiver mental health; family background information. The research committee then considered specific measures that should be included in a harmonised assessment protocol that adequately covers these domains. Each measure was reviewed against the minimum standards for patient‐reported outcome measures recommended by the International Society for Quality of Life Research (Reeve et al., 2013). See Table S5, for this information.

The measures included in the core and extended transdiagnostic assessment protocols are presented in Table 4. For a summary of the psychometric properties of each measure, as well as whether they are freely available for use, please see Table S5. The committee recommended that the core assessment protocol should be administered in full, and the additional measures within the extended assessment protocol administered where possible. Total completion time for the core transdiagnostic protocol is estimated to be between 45 and 60 min, while total completion time for the extended transdiagnostic protocol is expected to be between 100 and 140 min. An advantage of both assessment protocols is that they can be completed by caregivers online before they and their child attend their appointment. This is likely to be particularly important for successful uptake, given our recent findings in developmental clinics where substantially higher response rates were seen for online as opposed to paper completion (Patel et al., 2021). Moreover, both the core and extended protocols can be automatically scored before an individual presents at a service, providing clinicians and service providers with a snapshot of individuals, including areas of potential concern, before they arrive for assessment. We provide an overview of each domain in turn.

**TABLE 4:** Recommended measures for transdiagnostic data collection in developmental clinics

Domain	Core assessment protocol			Extended assessment protocol		
	Instrument	Duration	Reasons for inclusion	Instrument	Duration	Reasons for inclusion
Functioning and quality of life	EQ‐5D‐Y (Ravens‐Sieberer et al., 2010)	2 min	Provides ratings on health status across five dimensions.	Vineland Adaptive Behavior Scales, Third Edition – Domain Level form (Sparrow et al., 2016)	15–25 min	Widely used and endorsed for assessing daily adaptive functioning
	Child Health Utility 9D (Stevens, 2012)	2 min	Provides ratings on health‐related quality of life; allows calculation of QALYs for cost utility analyses			
	Paediatric Quality of Life Inventory (Varni et al., 2001)	5–10 min	Widely used as measure of functioning and quality of life			
	Ages and Stages Questionnaire, Third Edition (Squires & Bricker, 2009)	10‐15 min	Provides screen of early development in infants from one month of age			
Child mental health	Strengths and difficulties Questionnaire (Goodman, 1997)	5–10 min	Widely used and endorsed for measuring child behavioural problems	Child Behaviour Checklist (Achenbach & Rescorla, 2000, 2001)	15–20 min	Gold‐standard tool for measuring child's behaviour, competencies and problems; generates DSM‐oriented scales
Caregiver mental health	Depression Anxiety Stress Scale (Lovibond & Lovibond, 1995)	5 min	Widely used for measuring self‐reported depression, anxiety and stress	Adult Self‐Report (Achenbach & Rescorla, 2003)	15–20 min	Measures current adaptive functioning and problems; generates DSM‐oriented scales
	Adult ADHD Self‐Report Scale (Ustun et al., 2017)	2 min	Widely endorsed self‐report screen for adult ADHD			
	Caregiver Strain Questionnaire – Short Form (Brannan et al., 2012)	2 min	Widely used measure of objective and subjective internalised caregiver strain			
Family background information	Demographics and SES	10 min	Collects family demographic and SES information			
	Intervention history	5 min	Collects information on current and previous interventions and barriers to access			
Cognitive functioning				Behaviour Rating Inventory of Executive Function (Gioia et al., 2003, 2015)	10–15 min	Widely used for measuring executive functions within child's everyday environment

Abbreviations: ADHD, attention‐deficit hyperactivity disorder; DSM, Diagnostic and Statistical Manual of Mental Disorders; QALYs, quality‐adjusted life‐years; SES, socioeconomic status.

#### Child functioning and quality of life

Irrespective of the specific diagnosis, adaptive functioning and quality of life are core areas of impairment in neurodevelopmental disorders. The recommended measures were selected based on their ability to provide a snapshot of a child's functioning and quality of life. The key measure tapping child functioning and quality of life is the Paediatric Quality of Life Inventory (PEDS‐QL; Varni et al., 2001), which is available in both child and parent rated versions and which has been widely used in observational studies and clinical trials across a range of neurodevelopmental disorders including autism (Sheldrick et al., 2012), ADHD (Sciberras et al., 2011), cerebral palsy (Varni et al., 2006) and language disorders (Le et al., 2021; Nicola & Watter, 2015). As was noted by a member of the research committee, while the PEDS‐QL is designed as a measure of quality of life it is probably better thought of as a measurement of functioning when completed by a caregiver (Jonsson et al., 2017). Additionally, two measures commonly used in the clinical and economic evaluation of health care were selected, the EQ‐5D‐Y (Ravens‐Sieberer et al., 2010) and the Child Health Utility 9D (CHU9D; Stevens, 2012), with a vision of calculating quality‐adjusted life years (QALYs) to conduct cost utility analyses. For infants under two years of age, the Ages and Stages Questionnaire, Third Edition (ASQ‐3; Squires & Bricker, 2009) was selected, a commonly used developmental screening measure which provides details on child development from 1 month of age. It should be noted that the ASQ‐3 and the PEDS‐QL have licensing costs associated with their use. A more comprehensive measure of adaptive functioning, namely, the *Vineland Adaptive Behaviour Scale*, Third Edition—Domain Level Form (Sparrow et al., 2016), is included in the extended transdiagnostic assessment protocol.

#### Child mental health

It was noted that child mental health symptoms are not always routinely assessed when children with neurodevelopmental disorders present at hospital and community‐based clinics, despite these children being at increased risk for mental health conditions (Shaw et al., 2012). Thus, a common measure of mental health suitable across a wide range of neurodevelopmental disorders was deemed critical for inclusion in the transdiagnostic assessment protocol. The measure recommended for inclusion in the core transdiagnostic protocol, the Strengths and Difficulties Questionnaire (SDQ; Goodman, 1997), is brief, provides a snapshot of current behavioural, social and emotional problems experienced by children aged 2–18 years, and has been used widely in children with various neurodevelopmental disorders including autism (Salayev & Sanne, 2017), ADHD (Becker et al., 2006), and cerebral palsy (McMahon et al., 2020). Moreover, the SDQ has recently been recommended for widespread use across developmental assessment services (Jongeling & Roberts, 2018). A more detailed measure of child mental health applicable for children aged 1.5–18 years which provides an indication of whether the child is likely to meet DSM‐5 criteria for certain disorders, the Child Behaviour Checklist (CBCL; Achenbach & Rescorla, 2000, 2001), is recommended in the extended transdiagnostic assessment protocol.

#### Caregiver mental health

Feedback from developmental clinics revealed that caregiver mental health was a domain that over half of the researchers and clinicians surveyed would like to see prioritised, however it was largely unaddressed in the clinics represented. Collecting mental health data on caregivers nationwide should provide invaluable insight into the severity of symptoms experienced by caregivers, whether these symptoms tend to be shared across neurodevelopmental disorders, and what supports and pathways should be provided to caregivers to improve child and family outcomes. The research committee deemed it important to assess caregiver strain alongside mental health, given the considerable stressors that can be associated with caring for a child with a neurodevelopmental disorder. In a similar fashion to the child mental health domain, the measures selected for inclusion in the core transdiagnostic assessment protocol, the Caregiver Strain Questionnaire Short Form 7 (CGSQ‐SF7; Brannan et al., 2012), the Depression Anxiety and Stress Scale (DASS‐21; Lovibond & Lovibond, 1995) and the Adult ADHD Self‐Report Scale (ASRS‐5; Ustun et al., 2017), are brief and widely used in the literature. These measures provide an overview of caregiver strain, current mental health symptoms and screen for adult ADHD, which a member of the research committee noted was over‐represented in parents of children with neurodevelopmental disorders, in line with recent findings (Okyar & Görker, 2020; Wesseldijk, Dieleman, van Steensel, Bartels, et al., 2018). A more comprehensive measure of mental health, the Adult Self‐Report (ASR; Achenbach & Rescorla, 2003), is recommended in the extended transdiagnostic protocol. In addition to providing an overview of mental health symptom severity, the ASR provides information on adaptive functioning and substance use, as well as an indication of whether the respondent is likely to meet DSM‐5 criteria for mental health conditions.

#### Family background information

A common theme that emerged when considering recommendations from clinics was the need to consistently collect accurate and harmonised background information, such as socio‐demographic data, on families presenting to hospital and community‐based clinics. Specifically, it was noted that information pertaining to educational attainment and employment of caregivers, as well as family income, is not routinely collected, despite being considered a key domain on which to focus. Additionally, clinicians noted that an understanding of a child's intervention history, as well as caregiver intervention history, is valuable for understanding the interventions that are frequently used, and whether they are similarly used across different diagnoses. As there are no validated measures assessing socioeconomic background in Australia that have been used in neurodevelopmental populations, a set of questions was developed in consultation with experts in the field of equitable health service provision. These questions are based on data routinely collected across child development clinics, supporting their appropriateness for use in neurodevelopmental populations. Similarly, there are no validated measures assessing intervention history for both children and caregivers in the neurodevelopment space. As such, we developed a series of questions in consultation with clinicians and service providers who have expertise in the field of child and caregiver interventions, and who currently work with neurodevelopmental disorders. The questions pertaining to socioeconomic status and demographics, and to child and caregiver intervention history are in Appendixes S1 and S2, respectively.

#### Cognitive functioning (extended transdiagnostic protocol only)

When considering the measures routinely used by neurodevelopmental clinics, it was noted that assessments of cognitive functioning are commonly conducted, however there was no consistency in the assessment or measures used. Cognitive difficulties are commonly associated with poorer outcomes for children and adolescents with neurodevelopmental disorders (Einfeld et al., 2006), and it was noted that an assessment of cognitive functioning is crucial for identifying impairments and recommending appropriate referral pathways (Coghill, 2021). Given that cognitive functioning is typically best assessed using face‐to‐face measures, and one key aim of the transdiagnostic assessment protocol was to select questionnaire‐based measures that could be completed by caregivers at home, the decision was made not to include the domain of cognitive functioning in the core transdiagnostic assessment protocol. However, given that executive function has been shown to impact on outcome (Albert et al., 2018; Demetriou et al., 2019), and executive function difficulties are commonly seen across neurodevelopmental disorders (Dajani et al., 2016), a measure of everyday executive function, the Behavior Rating Inventory of Executive Function, Second Edition (BRIEF 2; Gioia et al., 2003, 2015), is recommended for use in the extended transdiagnostic assessment protocol.

## DISCUSSION

This paper describes a national consultative process that engaged researchers, clinicians, community organisations and consumers across neurodevelopmental disorders. The consultation process brought together participants' feedback and provided insights on the transdiagnostic needs of people with neurodevelopmental disorders and their families. This information was distilled, and a set of measures proposed by the national Neurodevelopment Australia research committee, to better harmonise data collection and to inform on transdiagnostic needs nationally. The set of proposed measures tap into the domains of child functioning/quality of life, child mental health, caregiver mental health, and family background information. It was noteworthy that while many researchers and clinicians agreed these domains were important for both research and patient care, and expressed a desire to collect such information, few groups were routinely collecting information across all domains.

By assessing functioning and quality of life in children with neurodevelopmental disorders, clinicians and researchers can obtain a high‐quality snapshot of transdiagnostic functioning and identify areas where interventions are required. This domain was considered critical to measure transdiagnostically as, across neurodevelopmental disorders, children commonly experience difficulties in social and educational functioning, including developing and maintaining social relationships with others (ABS, 2019; Mikami et al., 2019). The majority of children have difficulties with independence and require assistance in day‐to‐day activities, such as communication, mobility, self‐care and health care, which contributes to decreased quality of life (ABS, 2019).

As children with neurodevelopmental disorders are at increased risk for other mental health conditions (Shaw et al., 2012), a broad assessment of child mental health symptoms was deemed critical to improve evaluation of additional mental health needs across neurodevelopmental disorders and to facilitate research into addressing these mental health needs. Despite the higher utilisation of diagnosis specific health services, there is growing awareness of the barriers children and their families face when accessing general health care supports. For example, it has been identified that children with neurodevelopmental disorders and their families have difficulty accessing common community mental health supports that are widely available to the general public (Venville et al., 2015; Weise et al., 2019; Whittle et al., 2017). Assessing mental health when children first present to services would enable identification of needs and facilitate referral to community mental health supports as appropriate. Moreover, assessing child mental health transdiagnostically could help inform symptoms and behaviours that are shared across disorders, as opposed to specific to a single diagnosis.

Caregivers of children with neurodevelopmental disorders are at increased risk for stress and mental health conditions (Bromley et al., 2004; Masefield et al., 2020; Sawyer et al., 2010; Wesseldijk, Dieleman, van Steensel, Bleijenberg, et al., 2018). As such, it was considered critical that caregiver mental health be measured transdiagnostically. The mental health vulnerabilities common in caregivers have been shown to be associated with broader negative effects for the family, predicting poorer outcomes over time (Wesseldijk, Dieleman, van Steensel, Bleijenberg, et al., 2018). For example, parental stress appears predictive of therapy adherence across neurodevelopmental disorders, such that increased perceived parental stress predicts lower therapy adherence in children diagnosed with autism, ADHD and/or intellectual disability (Loader et al., 2019). Considering caregiver mental health as a transdiagnostic factor may elucidate how caregiver well‐being can influence functioning and development in children, irrespective of the specific diagnosis. Assessment of caregiver mental health may also lead to the development of transdiagnostic, evidence‐based interventions which could improve outcomes for the whole family.

The clinics and research centres surveyed within this paper provide care to a diverse range of families, including a considerable proportion from lower socioeconomic backgrounds. Additionally, the type of interventions accessed by these families are incredibly diverse. Given that families from low socio‐economic backgrounds are at increased likelihood of poor outcomes and face increased barriers when attempting to access services (Raouafi et al., 2018; Wesseldijk, Dieleman, van Steensel, Bartels, et al., 2018), it was deemed important to collect information on family demographics and socio‐economic status. Collection of this information can facilitate the development of pathways to help these families access the services they need. Further, a clearer understanding of the types of interventions used by both children and caregivers was also considered critical, given that interventions are not equally accessed across or within neurodevelopmental disorders (Khetani et al., 2017; Tan‐MacNeill et al., 2020; Wright et al., 2015). Collection of these data will likely inform which interventions are most accessible to families, whether certain interventions are more likely to be used by specific groups of individuals, and how we can modify services and increase access to ensure that interventions are accessible to all families who need them, regardless of diagnosis and socioeconomic background.

This transdiagnostic assessment protocol will be promoted and made available to researchers, clinicians and community organisations working in child neurodevelopment research and services across Australia, and potentially internationally, to collect core harmonised transdiagnostic data. These instruments can provide a useful screen for transdiagnostic needs, such as caregiver mental health, child mental health, and functioning supports, that might be needed. On their own, however, they will likely result in false positives and therefore should be used for guiding further clinical investigation and subsequent referral (Chamberlain et al., 2021; Coghill et al., 2021; Goodman et al., 2000; Havdahl et al., 2016). The measures have been selected by researchers and clinicians with expertise in the field, keeping in mind the time and financial constraints of busy hospital‐based clinics. Moreover, the measures selected assess key domains of need and concern, as informed by community and clinician engagement. The protocol described in this paper has received ethics approval (2020/ETH02923) and has capacity for national and international collaboration. The transdiagnostic assessment protocol is now being disseminated widely across our network, with members of Neurodevelopment Australia implementing it in their research and clinical services.

The assessment protocol proposed in this paper, and specifically the information collected within it, can be harnessed to provide individualised, needs‐based supports and information to families who access services, which may facilitate improved outcomes. For example, caregivers that report significant levels of depression as identified following completion of the protocol could be supported with online resources, psychoeducation and clinical pathways for care. This assessment protocol could therefore facilitate clinical care pathways that may not currently exist in services. Our team is currently working with participating services to co‐design streamlined assessments and efficient processes for data collection as well as support pathways and intervention resources that are provided to families and clinicians based on needs identified in the transdiagnostic assessment protocol. In addition, it is already established that these transdiagnostic factors, such as caregiver mental health, may moderate treatment response for children (Campbell et al., 2020; Wesseldijk, Dieleman, van Steensel, Bleijenberg, et al., 2018). The use of a standardised transdiagnostic set may thus be useful for supporting personalised intervention practices for both research and clinical practice.

The proposed harmonised data collection protocol will allow for the development of a large transdiagnostic dataset that can be administered to children with neurodevelopmental disorders and their families who present to hospital‐based and community services, as well as research centres around the country. While there are domains relevant across neurodevelopmental disorders that are beyond the scope of the transdiagnostic assessment protocol proposed here (e.g., motor and cognitive assessments, see England‐Mason, 2020; Ketelaar et al., 1998; Vaidya et al., 2020; Wilson et al., 2018), the measures in the proposed assessment protocol should add value to current clinical practice and research, as well as having the potential to inform policy making and practice. A summary of the potential value of the assessment protocol across these domains is shown in Table 5. Furthermore, we acknowledge that the measures in the assessment protocol are caregiver‐report questionnaires. However, many of these measures have self‐report versions available for older children, which will enable us to combine different reports (e.g., self‐ and caregiver‐report) as needed.

**TABLE 5:** Potential value of transdiagnostic assessment protocol across multiple domains

Domain	Potential value of assessment protocol
Clinical services	Provides structure to assessment and recording of information at the time of first presentation. Highlights and clarifies complexity in cases, and provides a clear baseline from which change can be measured. Enables clinical services to measure symptoms and areas of need, rather than focussing solely on an individual diagnosis. Allows clinicians to provide referrals and information resources based on transdiagnostic needs identified
Research	Facilitates national data collection in the neurodevelopment space and allows large‐scale research questions to be addressed. Provides opportunity to understand the suitability of these measures for culturally and linguistically diverse groups
	Enables data linkage on a national scale following participant consent. Data can be linked with other routinely collected data, including hospital admissions, NDIA service utilisations, the Medicare and pharmaceutical Benefits Schemes, the Juvenile Justice Minimum data Set, the National Assessment program for Literacy and Numeracy, and census data
Policy and Practice	National implementation of the assessment protocol would enable identification of the cross‐cutting needs of children with neurodevelopmental disorders and their families. This information could be used by policy makers to inform on national resource allocation and funding decisions. This transdiagnostic approach has potential to lead to positive downstream approaches to better support children at school, where a positive or negative experience can make an enormous contribution to mental health outcomes

Based on the data access and sharing policies that our group are currently preparing, and with ethics and governance approval across sites, individuals and groups who contribute data using the assessment protocol will have access to the data, and also to the expertise of the researchers and clinicians who have developed the protocol. This approach will facilitate data sharing and linkage, and future collaborative research opportunities. Given the difficulties in constructing a core set of measures that have been outlined when discussing the benefits and challenges of this type of integrative data analysis (Hussong et al., 2013), a major benefit of the proposed assessment protocol is that it comprises an agreed upon set of measures that will be consistently used by research centres and clinics nationally. Interested collaborators, including researchers and clinicians, are invited to contact the authors to discuss implementation of the protocol in their setting and can contribute to the dataset by using the transdiagnostic assessment protocol with their cohorts. This will promote a collaborative approach to data analysis, and will enable substantially increased statistical power, the potential to address research questions that cannot be answered by a single research centre or clinic, and importantly, the opportunity to develop a collective science of transdiagnostic assessment, where similarities and differences across clinical cohorts are evaluated. Data will be collected, stored and shared electronically on secure, University‐managed servers. Moreover, while the measures within the transdiagnostic assessment protocol were not selected based on their ability to track change or measure outcomes over time, our vision is to develop a complementary protocol designed to measure needs and outcomes, and monitor change over time. Participating children and families will be invited to complete this separate protocol, enabling us to conduct follow‐up assessments to gain insight into outcomes and change over time.

A long‐term goal of this transdiagnostic assessment protocol is to enable the development of investigator‐led, multi‐site, transdiagnostic research studies which can address the needs of many children and families, as opposed to single‐site, diagnosis‐specific studies, which are restricted to addressing the needs of a single population. Based on the services with which we are currently engaged, there is the potential for this assessment protocol to be administered to thousands of children per year, a sample which will increase as more research and clinical services utilise the protocol. Further, the research questions that could be addressed using data collected in this assessment protocol are numerous, with enormous potential for future research collaborations across universities and clinics. Possibilities for future work in this field range from an evaluation of the functioning levels and mental health needs of families presenting to services, to an evaluation of supports and information that could be provided to families based on needs identified upon completion of the transdiagnostic assessment protocol, to the development of transdiagnostic clinical trials. For example, this transdiagnostic assessment protocol may identify areas of need and concern that are common across diagnoses. This information could then be used to design a multi‐site intervention in which participants could enrol based on their symptom presentation, as opposed to their primary diagnosis. Such an approach would facilitate a departure from categorical approaches to the study of neurodevelopment and psychopathology and would enable acknowledgement of, and support for, the complexities and comorbidities that are commonly seen in the neurodevelopment space. Research has begun to consider how a transdiagnostic approach can be used to identify intervention targets suitable across disorders (Finlay‐Jones et al., 2019; Rigney et al., 2018), however implementation of a transdiagnostic framework in research and practice requires educational, cultural, and practical shifts.

While the disorders mentioned in this protocol paper are not all included under the heading of neurodevelopmental disorders in the DSM 5 or ICD 11, it should be noted that the DSM 5 and ICD 11 categorise neurodevelopmental disorders under the more general heading of mental disorders, which inherently results in more restrictive inclusion criteria. We believe a fundamental shift is required that enables neurodevelopmental assessment and intervention across diagnoses, particularly those that are associated with social, motor, and cognitive delays. The major gain from this shift is that focus can be placed on broad neurodevelopmental challenges and opportunities for each child while being agnostic to the diagnostic silos that tend to create barriers to accessing knowledge, service expertise and intervention opportunities that can improve long‐term developmental trajectories. It should also be recognised that when a child has significant delay in one domain (such as motor) they will be at far greater risk of delay in other domains (such as social and cognitive) and this interaction requires a more integrative framework to comprehensively address needs.

While the potential value and benefit of a harmonised transdiagnostic assessment protocol is clear and there is evidence that the families accessing these services would prefer to provide information online as opposed to on paper (Patel et al., 2021), we acknowledge that there are potential barriers to successful implementation. For instance, additional resources and support are required to ensure an assessment protocol of this sort is useful to all involved. While support and engagement from peak bodies and stakeholders is critical, adequate infrastructure is key to ensuring the successful implementation of a national transdiagnostic assessment protocol as proposed in this paper. An economic evaluation will be conducted from both a health system and societal perspective. This is essential to determine whether the costs to the service that are associated with implementation (e.g., infrastructure, resources) are feasible across clinics and if the benefits of the protocol (e.g., identification of transdiagnostic needs and subsequent referral to appropriate services) outweigh these implementation costs. Similarly, for successful implementation of this assessment protocol, it is important that state health departments support its implementation and help to facilitate the rollout across services where required. To enable ongoing evaluation of the transdiagnostic assessment protocol, we will use an implementation framework, assessing key implementation outcomes of acceptability, appropriateness, feasibility, adoption, costs, fidelity and sustainability (Proctor et al., 2011). This will allow for the essential evaluation of cost, successful implementation to new sites and national scalability.

Another potential barrier to implementation of this protocol lies in the cost of purchasing the assessment measures. In line with our aim to select measures in the public domain to ensure ease of access, the majority of measures selected for inclusion in the core assessment protocol are freely available and selected based on their ability to identify transdiagnostic needs. However, the measures included in the extended assessment protocol have varying degrees of licensing costs and registration requirements associated with their use. These measures were selected given their strong psychometric properties and ability to comprehensively assess the domains of interest. However, participating clinics must take these factors into consideration when deciding whether they have the resources to administer the extended assessment protocol.

The measures in this assessment protocol were selected by experts in the field following multiple consultations and using a transdiagnostic approach. Nevertheless, we note that the approach used to develop the transdiagnostic assessment protocol is not without its limitations. While the COS‐STAR guidelines were followed when developing this protocol, a more structured method of eliciting and synthesising stakeholder feedback may have enabled greater replicability by other groups. Further, despite the expertise of the research committee, we note that there was no external validation of the recommended assessment protocol through a large‐scale Delphi survey. In spite of these limitations, the transdiagnostic assessment protocol proposed here still has immense potential to harmonise data collection, which is often fragmented across services and conditions, and to provide much‐needed insight into the needs of children with neurodevelopmental disorders and their families.

In summary, while the science and application of transdiagnostic research and practice is in its infancy, this paper proposes a harmonised transdiagnostic assessment protocol which can be used by researchers, clinicians and community organisations to understand better the needs, concerns and vulnerabilities of children with neurodevelopmental disorders and their families. By engaging researchers, clinicians and community members across the field of neurodevelopmental disorders, a transdiagnostic assessment protocol has been developed, that has the potential to address the needs of children and families in the neurodevelopment space. This approach is the first step in facilitating nationwide research that is both directly informed by the community and is clinically relevant for families accessing hospital‐ and community‐based developmental services. The long‐term vision of this approach is to conduct research which will ultimately improve the lives of children with neurodevelopmental disorders and their families.

## CONFLICT OF INTERESTS

Nicole Rogerson and Phillipa Quinn are directors of Neurodevelopment Australia. Christel M. Middeldorp is a member of the Editorial Advisory Board for JCPP *Advances*. The remaining authors have declared that they have no competing or potential conflicts of interest. [Corrections made on 22 June 2022, after first online publication: This Conflict of Interests statement has been updated in this version.]

## ETHICS STATEMENT

The protocol described in this paper has received ethics approval from the Sydney Children's Hospital Network Human Research Ethics Committee (HREC Reference: 2020/ETH02923).

## Supporting information

Supporting Information S1

## Data Availability

Data sharing is not applicable to this article as no new data were created or analysed in this study.

## ENDNOTES

^1^
The University of Sydney, Brain and Mind Centre; Sydney Children's Hospital Network Randwick and Westmead campus Nepean Blue Mountains Local Health District, Nepean Hospital; The University of New South Wales; Monash University, Turner Institute for Brain and Mental Health; The Royal Children's Hospital Melbourne; The University of Melbourne; Children's Health Queensland Hospital and Health Service; The University of Queensland; Macquarie University; Deakin University, Deakin Child Study Centre; The University of Western Australia, Telethon Kids Institute; Murdoch Children's Research Institute.

^2^
ADHD Australia; ADHD Foundation; ADHD United; ADHD Western Australia; Amaze; Autism Awareness Australia; Autism Parents Australia; Autism Spectrum Australia; Cerebral Palsy Alliance; The Canberra & Queanbeyan ADD Support Group; The Growing Space.
