# Supplementary material for: A national harmonised data collection network for neurodevelopmental disorders: A transdiagnostic assessment protocol for neurodevelopment, mental health, functioning and well‐being
Source: JCPP Adv. 2021 Nov 26;1(4):e12048. doi: 10.1002/jcv2.12048 (PMC10242941; doi:10.1002/jcv2.12048)
Supplement: Supplementary file 1 — Supporting Information S1 [file JCV2-1-e12048-s001.docx]

**Table S1**

*COS-STAR Guidelines.*

| **Item No.** | **Recommendation** | **Brief description of how each item was reported** |
| --- | --- | --- |
| **Title/Abstract** | | |
| 1a | Identify in the title that the paper reports the development of a COS | Title states that paper reports the development of a transdiagnostic assessment protocol. |
| 1b | Provide a structured summary | Structured abstract summarises the context for the development of the assessment protocol, the process of development, and implications of the assessment protocol for healthcare and research. |
| **Introduction** | | |
| 2a | Describe the background and explain the rationale for developing the COS | Background and rationale for the development of the transdiagnostic assessment protocol is described throughout the introduction. |
| 2b | Describe the specific objectives with reference to developing a COS | There are no data collection protocols that are integrated with clinical services and that can collect standardised information from children with neurodevelopmental disorders and their families.  The objective of this paper was to collaborate with a national consortium of researchers, clinicians, and community representatives in order to propose an assessment protocol for harmonised data collection across research centres, hospital-based and community clinics in the field of child neurodevelopment. |
| 3a | Describe the health condition(s) and population(s) covered by the COS | The transdiagnostic assessment protocol was developed for use in children with neurodevelopmental disorders and their families. |
| 3b | Describe the intervention(s) covered by the COS | N/A – this paper describes the development of an assessment battery, not an outcome set intended for use in clinical trials. |
| 3c | Describe the setting(s) in which the COS is to be applied | The assessment protocol is designed to be applied in researcher centres, hospital-based and community clinics. |
| **Methods** | | |
| 4 | Indicate where the COS development protocol can be accessed, if available and/or the study registration details | A protocol is not available, and the study was not registered. |
| 5 | Describe the rationale for stakeholder groups involved in the COS development process, eligibility criteria for participants from each group and a description of how the individuals involved were identified | Information pertaining to groups of individuals involved in the development of the assessment protocol (researchers, clinicians, community representatives, research committee) are described in the method section. |
| 6a | Describe the information sources used to identify an initial list of outcomes | Information sources constituted information available in the literature and information received from researchers, clinicians and community representatives. |
| 6b | Describe how outcomes were dropped/combined, with reasons (if applicable) | Specific measures were combined into domains. Criteria for selecting specific measures is described in the method section. |
| 7 | Describe how the consensus process was undertaken | The technique used to reach consensus is described in the method section. |
| 8 | Describe how outcomes were scored and scores summarised | Outcomes were decided upon following discussion. |
| 9a | Describe the consensus definition | Consensus that a measure should be included was based upon agreement amongst all measures of the research committee. |
| 9b | Describe the procedure for determining how outcomes were included or excluded from consideration during the consensus process | The criteria used to select measures for inclusion in the assessment protocol is described in the method section. |
| 10 | Provide a statement regarding the ethics and consent issues for the study | A statement outlining the ethical approval received for this study is provided in the discussion. |
| **Results** | | |
| 11 | Describe any changes from the protocol (if applicable), with reasons, and a describe what impact these changes have on the results | N/A |
| 12 | Present data on the number and relevant characteristics of the people involved at all stages of COS development | Information pertaining to individuals involved (researchers, clinicians, community representative, research committee) is provided in the results section. |
| 13a | List all outcomes considered at the start of the consensus process | All measures provided by stakeholders are described in Supplementary Table 3. |
| 13b | Describe any new outcomes introduced and any outcomes dropped, with reasons, during the consensus process | The process of selecting measures, and detailed descriptions of these measures are described in the results section. |
| 14 | List the outcomes in the final core outcome set | Table 4 describes the measures in the assessment protocol. |
| **Discussion** | | |
| 15 | Discuss any limitations in the COS development process | Limitations in the development of the assessment protocol are stated in the discussion. |
| 16 | Provide an interpretation of the final COS in the context of other evidence, and implications for future research | The discussion provides details of how the assessment protocol can be made available to researchers, clinicians and community organisations in the field of child neurodevelopment, where there are currently no standardised data collection protocols with transdiagnostic relevance.  The potential future value of the assessment protocol across multiple domains, including clinical services, research, policy and practice, is described throughout the discussion and in Table 5. |
| **Other information** | | |
| 17 | Describe sources of funding, role of funders | This work was supported by the Brain and Mind Centre Child Neurodevelopment and Mental Health team. |
| 18 | Describe any conflicts of interest within the study team and how these were managed | A conflict of interest disclosure statement is included. |

**Table S2**

*Australian Public Developmental Clinics Surveyed – Age Range of Children seen per Clinic Speciality.*

| Clinic specialty | Age range of patients (years) |
| --- | --- |
| Movement disorders | 2 – 18 |
| Cerebral palsy | 0 – 18 |
| Early developmental signs^1^ | 0 – 3.5 |
| Developmental assessment^2^ | 0 – 18 |
| Eating Disorders | 7 – 18 |
| Autism | 0 – 18 |
| ADHD | 3 – 18 |
| Neuromuscular disorders | 0 – 18 |
| Epilepsy | 0 – 16 |

*Note:* ^1^Early developmental signs – patients are children who were in the neonatal intensive care unit. Patients are seen routinely until 3.5 years of age. ^2^Developmental assessment – patients are children who have been referred to an assessment clinic with a suspected developmental delay requiring evaluation. Abbreviations: ADHD, attention-deficit hyperactivity disorder.

**Table S3**

*Australian Public Developmental Clinics Surveyed – Assessments and Measures used in each Clinic Speciality, Clustered by Broad Domains.*

| **Clinic Speciality** | **Diagnostic Interview/Medical Assessment** | **Symptom/Behaviour Screen** | **Cognitive/Developmental Assessment** | **Adaptive Functioning** | **Parental Screen** | **Motor Functioning** |
| --- | --- | --- | --- | --- | --- | --- |
| Movement disorders | Diagnostic interview including family history | Children’s Yale-Brown Obsessive Compulsive Scale (CY-BOCS; Scahill et al., 1997) |  | Children’s Global Assessment Scale (C-GAS; Shaffer et al., 1983) | Depression Anxiety Stress Scales – Short Form (DASS-21; Lovibond & Lovibond, 1995) |  |
|  | Kiddie Schedule for Affective Disorders and Schizophrenia (K-SADS; Kaufman et al., 1997) | Conners Third Edition (Conners 3; Conners & Goldstein, 2009) |  |  |  |  |
|  | Neurological exam | Child Behavior Checklist (CBCL; Achenbach & Rescorla, 2000, 2001) |  |  |  |  |
|  |  | Yale Global Tic Severity Scale (YGTSS; Leckman et al., 1989) |  |  |  |  |
|  |  | Swanson, Nolan, and Pelham Rating Scale (SNAP-IV; Swanson, Lerner, March, & Gresham, 1999) |  |  |  |  |
|  |  | The Screen for Child Anxiety Related Disorders (SCARED; Birmaher et al., 1997) |  |  |  |  |
|  |  | University of Sao Paulo Sensory Phenomena Scale (USP-SPS; Rosario et al., 2009) |  |  |  |  |
|  |  | Child and Adolescent Trauma Screen (CATS; Sachser et al., 2017) |  |  |  |  |
|  |  | Parent Tic Questionnaire (PTQ; Chang et al., 2009) |  |  |  |  |
|  |  | Child Obsessive Compulsive Disorder Impact Scale – Revised (COIS-R; Piacentini, Peris, Bergman, Chang, & Jaffer, 2007) |  |  |  |  |
|  |  | Family Accommodation Scale for Obsessive-Compulsive Disorder Self-Rated Version (FAS-SR; Pinto, Van Noppen, & Calvocoressi, 2013) |  |  |  |  |
|  |  | Obsessive Compulsive Inventory – Child Version (OCI-CV; Foa et al., 2010) |  |  |  |  |
|  |  | Behavior Rating Inventory of Executive Functioning (BRIEF; Gioia, Espy, & Isquith, 2003; Gioia, Isquith, Guy, & Kenworthy, 2015) |  |  |  |  |
|  |  | Strengths and Difficulties Questionnaire (SDQ; Goodman, 1997) |  |  |  |  |
| Cerebral palsy | Physical exam | Communication Function Classification System (CFCS; Hidecker et al., 2011) |  |  |  | Gross Motor Function Classification System (GMFCS; Palisano, Rosenbaum, Bartlett, Livingston, & Neurology, 2008) |
|  |  |  |  |  |  | Manual Ability Classification System (MACS; Eliasson et al., 2006) |
|  |  |  |  |  |  | Functional Mobility Scale (FMS; Graham, Harvey, Rodda, Nattrass, & Pirpiris, 2004) |
|  |  |  |  |  |  | Hypertonia Assessment Tool (HAT; Jethwa et al., 2010) |
| Early developmental signs^1^ | Diagnostic interview including family history. |  | Multidisciplinary Developmental Assessment |  |  | General Movements Assessment [25] |
|  | Hammersmith Infant Neurological Exam [26] |  | Bayley Scales of Infant and Toddler Development, Third Edition [27] |  |  |  |
| Developmental assessment^2^ | Diagnostic interview including family history | Conners Third Edition (Conners 3; Conners & Goldstein, 2009) | Wechsler Preschool and Primary Scale of Intelligence – Fourth Edition (WPPSI-IV; Wechsler, 2012) | Adaptive Behaviour Assessment System – Third Edition (ABAS-3; Harrison & Oakland, 2005) |  |  |
|  | Physical exam/Medical assessment | Child Behavior Checklist (CBCL; Achenbach & Rescorla, 2000, 2001) | Wechsler Intelligence Scale for Children (WISC-V; Wechsler, 2014) | Vineland Adaptive Behaviour Scales, Third Edition (Vineland-3; Sparrow, Cicchetti, & Saulnier, 2016) |  |  |
|  | Autism Diagnostic Interview-Revised (ADI-R; Lord, Rutter, & Le Couteur, 1994) | Vanderbilt ADHD Diagnostic Rating Scale (VADRS; Wolraich et al., 2003) | Griffiths Scales of Child Development, Third Edition (Griffiths III; Green et al., 2016) |  |  |  |
|  |  | Modified Checklist for Autism in Toddlers (M-Chat; Robins, Fein, Barton, & Green, 2001) | Autism Diagnostic Observation Schedule, Second Edition (ADOS-2; Lord et al., 2008) |  |  |  |
|  |  | Childhood Autism Rating Scale (CARS; Schopler, Reichler, & Renner, 2010) | Bayley Scales of Infant and Toddler Development, Third Edition [27] |  |  |  |
|  |  | Yale Global Tic Severity Scale (YGTSS; Leckman et al., 1989) | Mullen Scales of Early Learning [38] |  |  |  |
|  |  | Canadian ADHD Resource Alliance (CADDRA) ADHD Checklist [39] | Stanford-Binet Intelligence Scales, Fifth Edition (SB-5; Roid, 2003) |  |  |  |
|  |  | Ages & Stages Questionnaire, Third Edition (ASQ-3; Squires & Bricker, 2009) | Wechsler Individual Achievement Test, Third Edition (WIAT-III; Wechsler, 2009) |  |  |  |
|  |  | Spence Children’s Anxiety Scale (SCAS; Spence, 1998) | Teach of Everyday Attention for Children, Second Edition (TEA-Ch2; Manly, Anderson, Crawford, George, & Roberson, 2016) |  |  |  |
|  |  | Repetitive Behaviour Scale, Revised (RBS-R; Lam & Aman, 2007) | NEPSY-II [46] |  |  |  |
|  |  | Aberrant Behavior Checklist (ABC; Aman, Singh, Stewart, & Field, 1985) | Clinical Evaluation of Language Fundamentals, Fifth Edition (CELF-5; Semel, Wiig, & Secord, 2013) |  |  |  |
|  |  | Developmental Behaviour Checklist (DBC; Einfeld & Tonge, 1995) | Children’s Communication Checklist – Second Edition (CCC-2; Bishop, 2012) |  |  |  |
|  |  | Strengths and Difficulties Questionnaire (SDQ; Goodman, 1997) |  |  |  |  |
|  |  | Behavior Rating Inventory of Executive Functioning (BRIEF; Gioia, Espy, & Isquith, 2003; Gioia, Isquith, Guy, & Kenworthy, 2015) |  |  |  |  |
|  |  | Social Communication Questionnaire (SCQ; Berument, Rutter, Lord, Pickles, & Bailey, 1999) |  |  |  |  |
|  |  | Social Responsiveness Scale, Second Edition (SRS-2; Constantino & Gruber, 2012) |  |  |  |  |
|  |  | Short Sensory Profile 2 (SSP-2; McIntosh, Miller, Shyu, & Dunn, 1999) |  |  |  |  |
| Eating Disorders | Eating Disorder Examination (EDE; Fairburn, Cooper, & O’Connor, 1993) | Eating Disorder Examination Questionnaire (EDE-Q; Fairburn & Beglin, 1994) |  |  |  |  |
|  |  | Children’s Yale-Brown Obsessive Compulsive Scale (CY-BOCS; Scahill et al., 1997) |  |  |  |  |
|  |  | Revised Children’s Anxiety and Depression Scale (RCADS; Chorpita, Yim, Moffitt, Umemoto, & Francis, 2000) |  |  |  |  |
| Autism | Diagnostic interview including family history | Autism Spectrum Quotient (AQ; Baron-Cohen, Wheelwright, Skinner, Martin, & Clubley, 2001) | Autism Diagnostic Observation Schedule, Second Edition (ADOS-2; Lord et al., 2008) | Vineland Adaptive Behaviour Scales, Third Edition (Vineland-3; Sparrow et al., 2016) | Depression Anxiety Stress Scales – Short Form (DASS-21; Lovibond & Lovibond, 1995) | Peabody Development Motor Scales – 2 (PDMS-2; Folio & Fewell, 2000) |
|  | Developmental, Dimensional and Diagnostic Interview (3Di; Skuse et al., 2004) | Children’s Yale-Brown Obsessive Compulsive Scale (CY-BOCS; Scahill et al., 1997) | Mullen Scales of Early Learning [38] | Children’s Global Assessment Scale (C-GAS; Shaffer et al., 1983) | Caregiver Strain Questionnaire (CSQ; Brannan, Heflinger, & Bickman, 1997) | Movement Assessment Battery for Children (M-ABC; Henderson, Sugden, & Barnett, 2007) |
|  | Development and Well-Being Assessment (DAWBA; Goodman, Ford, Richards, Gatward, & Meltzer, 2000) | Conners Third Edition (Conners 3; Conners & Goldstein, 2009) | Children’s Communication Checklist – Second Edition (CCC-2; Bishop, 2012) | WHO Disability Assessment Schedule (WHODAS 2.0; Ustun, Kostanjsek, Chatterji, & Rehm, 2010) | Hamilton Depression Rating Scale (HAM-D; Hamilton, 1960) |  |
|  | Autism Diagnostic Interview-Revised (ADI-R; Lord et al., 1994) | Child Behavior Checklist (CBCL; Achenbach & Rescorla, 2000, 2001) | Communication and Symbolic Behaviour Scales Developmental Profile (CSBS-DP Wetherby & Prizant, 2002) | Child Health and Illness Profile (CHIP; Riley et al., 2004) | Conners’ Adult ADHD Rating Scale (CAARS; Conners, Erhardt, & Sparrow, 1998) |  |
|  |  | Yale Global Tic Severity Scale (YGTSS; Leckman et al., 1989) | Clinical Evaluation of Language Fundamentals, Fifth Edition (CELF-5; Semel et al., 2013) |  |  |  |
|  |  | Repetitive Behaviour Scale, Revised (RBS-R; Lam & Aman, 2007) | McArthur-Bates Communicative Development Inventories (MCDI; Dale, 1991) |  |  |  |
|  |  | Aberrant Behavior Checklist (ABC; Aman et al., 1985) | Wechsler Preschool and Primary Scale of Intelligence – Fourth Edition (WPPSI-IV; Wechsler, 2012) |  |  |  |
|  |  | Developmental Behaviour Checklist (DBC; Einfeld & Tonge, 1995) | Wechsler Intelligence Scale for Children (WISC-V; Wechsler, 2014) |  |  |  |
|  |  | PDD Behaviour Inventory Screening Version (PDDBI-SV; Cohen, Schmidt-Lackner, Romanczyk, & Sudhalter, 2003) |  |  |  |  |
|  |  | Kessler Psychological Distress Scale (K10; Kessler et al., 2002) |  |  |  |  |
|  |  | Strengths and Difficulties Questionnaire (SDQ; Goodman, 1997) |  |  |  |  |
|  |  | Behavior Rating Inventory of Executive Functioning (BRIEF; Gioia, Espy, & Isquith, 2003; Gioia, Isquith, Guy, & Kenworthy, 2015) |  |  |  |  |
|  |  | Social Communication Questionnaire (SCQ; Berument et al., 1999) |  |  |  |  |
|  |  | Spence Children’s Anxiety Scale (SCAS; Spence, 1998) |  |  |  |  |
|  |  | Social Responsiveness Scale, Second Edition (SRS-2; Constantino & Gruber, 2012) |  |  |  |  |
|  |  | Short Sensory Profile 2 (SSP-2; McIntosh et al., 1999) |  |  |  |  |
|  |  | Health of the Nation Outcome Scales – Children and Adolescents (HoNOSCA; Gowers et al., 1999) |  |  |  |  |
|  |  | Quantitative Checklist for Autism in Toddlers (Q-CHAT; Allison et al., 2008) |  |  |  |  |
|  |  | SWAN Rating Scale for ADHD [73] |  |  |  |  |
| ADHD | Diagnostic interview including family history | Conners Third Edition (Conners 3; Conners & Goldstein, 2009) | Wechsler Preschool and Primary Scale of Intelligence – Fourth Edition (WPPSI-IV; Wechsler, 2012) | Adaptive Behaviour Assessment System – Third Edition (ABAS-3; Harrison & Oakland, 2005) | Conners’ Adult ADHD Rating Scale (CAARS; Conners, Erhardt, & Sparrow, 1998) |  |
|  | Developmental, Dimensional and Diagnostic Interview (3Di; Skuse et al., 2004) | Child Behavior Checklist (CBCL; Achenbach & Rescorla, 2000, 2001) | Wechsler Intelligence Scale for Children (WISC-V; Wechsler, 2014) | Vineland Adaptive Behaviour Scales, Third Edition (Vineland-3; Sparrow et al., 2016) | Adult Self Report (ASR; Achenbach & Rescorla, 2003) |  |
|  | Development and Well-Being Assessment (DAWBA; Goodman et al., 2000) | Vanderbilt ADHD Diagnostic Rating Scale (VADRS; Wolraich et al., 2003) | Wechsler Individual Achievement Test, Third Edition (WIAT-III; Wechsler, 2009) | Child Health and Illness Profile (CHIP; Riley et al., 2004) |  |  |
|  |  | Spence Children’s Anxiety Scale (SCAS; Spence, 1998) | Teach of Everyday Attention for Children, Second Edition (TEA-Ch2; Manly et al., 2016) |  |  |  |
|  |  | Aberrant Behavior Checklist (ABC; Aman et al., 1985) | NEPSY-II [46] |  |  |  |
|  |  | Strengths and Difficulties Questionnaire (SDQ; Goodman, 1997) | Wide Range Assessment of Memory & Learning, Second Edition (WRAML2; Sheslow & Adams, 2003) |  |  |  |
|  |  | SWAN Rating Scale for ADHD [73] | Wide Range Assessment of Visual Motor Abilities (WRAVMA; Adams & Sheslow, 1995) |  |  |  |
|  |  | Behavior Rating Inventory of Executive Functioning (BRIEF; Gioia, Espy, & Isquith, 2003; Gioia, Isquith, Guy, & Kenworthy, 2015) | Clinical Evaluation of Language Fundamentals, Fifth Edition (CELF-5; Semel et al., 2013) |  |  |  |
|  |  | Autism Spectrum Quotient (AQ; Baron-Cohen et al., 2001) | Children’s Communication Checklist – Second Edition (CCC-2; Bishop, 2012) |  |  |  |
|  |  |  |  |  |  |  |
| Neuromuscular disorders | Diagnostic interview including family history. |  |  |  |  |  |
| Epilepsy | Engel Epilepsy Surgery Outcome Scale [77] |  | Griffiths Scales of Child Development, Third Edition (Griffiths III; Green et al., 2016) |  |  |  |
|  |  |  | Bayley Scales of Infant and Toddler Development, Third Edition [27] |  |  |  |

*Note:* ^1^Early developmental signs – patients are children who were in the neonatal intensive care unit. Patients are seen routinely until 3.5 years of age. ^2^Developmental assessment – patients are children who have been referred to an assessment clinic with a suspected developmental delay requiring evaluation. Abbreviations: ADHD, attention-deficit hyperactivity disorder.

**Table S4**

*Australian Public Developmental Clinics Surveyed – Assessments and Measures Recommended for Use by each Clinic Speciality.*

| **Clinic Speciality** | **Functioning/Quality of Life** | **Family Background Information** | **Caregiver Mental Health** | **Child Behaviour/Symptoms** | **Cognitive Functioning/ Child Development** |
| --- | --- | --- | --- | --- | --- |
| Movement disorders | Measure of functioning at school | Socio-Economic Indexes for Areas (SEIFA; Australian Bureau of Statistics, 2016) | Measure of parental/caregiver stress and mental health | Measure of child emotional wellbeing | Developmental screening tools |
|  | Health of the Nation Outcome Scales – Children and Adolescents (HoNOSCA; Gowers et al., 1999) | Socio-economic determinants | Depression Anxiety Stress Scales – Short Form (DASS-21; Lovibond & Lovibond, 1995) | Conners Third Edition (Conners 3; Conners & Goldstein, 2009) |  |
|  |  |  |  | Kiddie Schedule for Affective Disorders and Schizophrenia (K-SADS; Kaufman et al., 1997) |  |
|  |  |  |  | Swanson, Nolan, and Pelham Rating Scale (SNAP-IV; Swanson et al., 1999) |  |
|  |  |  |  | Child and Adolescent Trauma Screen (CATS; Sachser et al., 2017) |  |
|  |  |  |  | Child Behavior Checklist (CBCL; Achenbach & Rescorla, 2000, 2001) |  |
|  |  |  |  | Strengths and Difficulties Questionnaire (SDQ; Goodman, 1997) |  |
| Cerebral palsy | Functional Mobility Scale (FMS; Graham et al., 2004) |  |  |  |  |
|  | Functional Independence Measure for Children (WeeFIM; Msall et al., 1994) |  |  |  |  |
|  | Functional Independence Measure (FIM; Linacre, Heinemann, Wright, Granger, & Hamilton, 1994) |  |  |  |  |
| Early developmental signs^1^ |  | Socio-Economic Indexes for Areas (SEIFA; Australian Bureau of Statistics, 2016) |  |  |  |
|  |  | Data on parental education |  |  |  |
| Developmental assessment^2^ | Pediatric Quality of Life Inventory (PedsQL; Varni, Seid, & Kurtin, 2001) | Measure of socio-economic stress | Measure of parental/caregiver stress and mental health | Measure of child emotional wellbeing | Preschool Language Scales, Fifth Edition (PLS-5; Zimmerman, Steiner, & Pond, 2012) |
|  | The Family Quality of Life Scale (FQOL; Hoffman, Marquis, Poston, Summers, & Turnbull, 2006) | Standardised Medical Data | Depression Anxiety Stress Scales – Short Form (DASS-21; Lovibond & Lovibond, 1995) | Developmental Behaviour Checklist (DBC; Einfeld & Tonge, 1995) | Clinical Evaluation of Language Fundamentals, Fifth Edition (CELF-5; Semel et al., 2013) |
|  | Vineland Adaptive Behaviour Scales, Third Edition (Vineland-3; Sparrow et al., 2016) | Diagnostic Classification Information |  | Child Behavior Checklist (CBCL; Achenbach & Rescorla, 2000, 2001) | Wechsler Individual Achievement Test, Third Edition (WIAT-III; Wechsler, 2009) |
|  |  |  |  | Strengths and Difficulties Questionnaire (SDQ; Goodman, 1997) | Comprehensive Developmental Assessment |
| Eating Disorders | Common measure of functional impairment |  |  | Measure of child emotional wellbeing |  |
|  | Common measure of family functioning |  |  |  |  |
| Autism | Measure of functioning aligned with International Classification of Functioning, Disability and Health (ICF) framework [84] | Socio-Economic Indexes for Areas (SEIFA; Australian Bureau of Statistics, 2016) | Measure of parental/caregiver stress and mental health | Measure of social isolation |  |
|  |  | Measure of financial strain | Depression Anxiety Stress Scales – Short Form (DASS-21; Lovibond & Lovibond, 1995) | Strengths and Difficulties Questionnaire (SDQ; Goodman, 1997) |  |
| ADHD | Measure of functioning | Measure capturing demographics, educational attainment, employment, family income | Measure of parental/caregiver stress and mental health | Measure of child emotional wellbeing |  |
|  |  |  | Adult Self Report (ASR; Achenbach & Rescorla, 2003) | Strengths and Difficulties Questionnaire (SDQ; Goodman, 1997) |  |
|  |  |  | Depression Anxiety Stress Scales – Short Form (DASS-21; Lovibond & Lovibond, 1995) | Child Behavior Checklist (CBCL; Achenbach & Rescorla, 2000, 2001) |  |
| Neuromuscular disorders | Measure of functioning | Measure of socio-economic stress | Measure of parental/caregiver stress and mental health |  |  |
|  |  |  | Depression Anxiety Stress Scales – Short Form (DASS-21; Lovibond & Lovibond, 1995) |  |  |
| Epilepsy |  |  |  |  |  |

**Table S5**

*Availability and Psychometric Performance of Selected Measures Based on the International Society for Quality of Life Research Minimum Standards for Patient-Reported Outcome Measures*

|  | Feasibility and acceptability | | | | | | Psychometric performance | |
| --- | --- | --- | --- | --- | --- | --- | --- | --- |
| *Instrument* | *Age range (years)* | *Freely available?* | *Respondent burden (i.e., number of items, approximate duration to complete)* | *Interpretability of scores (i.e., do users know what high or low scores represent?)* | *Prior use with neurodevelopmental disorders* | *Translated (i.e., >1 language version available* | *Reliability* | *Validity* |
| EQ-5D-Y [85] | 4 – 18 | Yes, when used for academic/non-commercial purposes. | 5 items.  2 minutes. | Yes – responses to each question result in a unique health state (from no problems across dimensions, to a lot of problems across dimensions). Health states can be converted to a single index value, reflecting how good or bad health state is based on country norms. | Yes (e.g., [86-88]). | Yes | Satisfactory test-retest reliability [85, 89]. | Satisfactory construct validity [90].  Satisfactory convergent validity [85, 89]. |
| Child Health Utility 9D [91] | 6 – 17 | Yes, when used for academic/non-commercial purposes. | 9 items.  2 minutes. | Yes – increasing scores refer to increasing levels of severity/impairment. Responses converted to utility scores using available algorithms. | Yes (e.g., [86, 87]). | Yes | Satisfactory internal consistency [92]. | Satisfactory convergent validity [89, 92].  Satisfactory discriminant validity [93]. |
| Pediatric Quality of Life Inventory [81] | 2 – 18 | Yes, for not funded academic research.  Licensing agreements apply for funded academic and organisational research. | 23 items.  5 – 10 minutes. | Yes – all items are reverse-scored, with higher scores indicating between quality of life. | Yes (e.g., [94-97] | Yes | Satisfactory internal consistency [90, 98, 99].  Satisfactory test-retest reliability [90]. | Satisfactory construct validity [90, 98, 99].  Satisfactory discriminant validity [99].  Satisfactory convergent validity [99]. |
| Ages and Stages Questionnaire, Third Edition [41] | 0 – 5.5 | No, one-off purchase of $229.84 USD allows copying of master questionnaires and scoring sheets. | 40 items.  10 – 15 minutes. | Yes – scores for each domain are compared to established cut-off points that indicate risk of developmental delay. | Yes (e.g., [100-102]) | Yes | Satisfactory internal consistency [41, 103].  Satisfactory test-retest reliability [41]. | Satisfactory concurrent validity [104].  Satisfactory construct validity [103]. |
| Strengths and Difficulties Questionnaire [19] | 2 – 17 | Yes. | 25 items.  5 – 10 minutes. | Yes – higher scores indicate increased difficulties. | Yes (e.g., [105, 106]). | Yes | Satisfactory internal consistency [107, 108].  Satisfactory test-retest reliability [108-110]. | Satisfactory concurrent validity [108, 109].  Satisfactory construct validity [108]. |
| Depression Anxiety Stress Scale [3] | 14 + | Yes. | 21 items.  5 minutes. | Yes – higher scores indicate increased depression, anxiety and stress symptoms. | N/A – is being completed by caregivers in transdiagnostic assessment protocol. | Yes | Satisfactory internal consistency [111-114]. | Satisfactory convergent validity [112-114].  Satisfactory divergent validity [112, 113]. |
| Adult ADHD Self-Report Scale [115] | 18 + | Yes. | 6 items.  2 minutes. | Yes – higher scores indicate increased symptoms. | N/A – is being completed by caregivers in transdiagnostic assessment protocol. | Yes | Satisfactory internal consistency [116, 117].  Satisfactory test-retest reliability [116]. | Satisfactory criterion validity [117]. |
| Caregiver Strain Questionnaire – Short Form [118] | 18 + | Yes, with author permission. | 7 items.  2 minutes. | Yes – higher scores indicate increased strain. | N/A – is being completed by caregivers in transdiagnostic assessment protocol. | Yes | Satisfactory internal consistency [118]. | Satisfactory construct validity [118]. |
| Vineland Adaptive Behavior Scales, Third Edition – Domain Level form [31] | 3 – 90 | No, must be purchased from publisher. Requires specific qualifications to administer and interpret. | 180 items.  15 – 25 minutes. | Yes – increased scores indicate higher functioning. Raw scores converted to a range of derived scores, including standard scores based on population norms and percentile ranks. | Yes (e.g., [119-121]) | Yes | Satisfactory internal consistency [122]. | Satisfactory construct validity [31].  Satisfactory convergent validity [31]. |
| Child Behavior Checklist [6, 7] | 1.5 – 18 | No, must be purchased from publisher. Requires specific qualifications to administer and interpret. | 99 items (1.5 – 5 years).  118 items (6 – 18 years).  15 – 20 minutes. | Yes – higher scores indicate increased severity. Raw scores converted to scale scores based on norms. | Yes (e.g., [123-125]) | Yes | Satisfactory internal consistency [126]. | Satisfactory convergent validity [126].  Satisfactory discriminant validity [126].  Satisfactory structural validity [127]. |
| Adult Self Report [74] | 18 – 59 | No, must be purchased from publisher. Requires specific qualifications to administer and interpret. | 126 items.  15 – 20 minutes. | Yes – higher scores indicate increased severity. Raw scores converted to scale scores based on norms. | N/A – is being completed by caregivers in transdiagnostic assessment protocol. | Yes | Satisfactory internal consistency [74, 128].  Satisfactory test-retest reliability [74]. | Satisfactory content validity [74].  Satisfactory criterion-related validity [74].  Satisfactory construct validity [74]. |
| Behavior Rating Inventory of Executive Function [17, 18] | 2 – 18 | No, must be purchased from publisher. Requires specific qualifications to administer and interpret. | 63 items.  10 – 15 minutes. | Yes – higher scores indicate increased executive functioning difficulties. Raw scores converted to T scores based on norms. | Yes (e.g., [129-133]). | Yes | Satisfactory internal consistency [134, 135].  Satisfactory test-retest reliability [135]. | Satisfactory discriminant validity [136].  Satisfactory factorial validity [134].  Satisfactory convergent validity [133]. |

*Note:* The Socioeconomic Status and Demographic Questionnaire and the Intervention History Questionnaire are not listed here as they are newly developed measures.

**Appendix S1**

*Socioeconomic Status and Demographics Questionnaire*

1. What is today's date?

Please use format dd/mm/yyyy _ _ / _ _ / _ _ _ _

1. What is your child’s gender?

- Male
- Female
- Other

1. What is your child’s date of birth?

Please use format dd/mm/yyyy _ _ / _ _ / _ _ _ _

1. If your child goes to school, where does your child go to school? Please choose one option.

- Not relevant - my child does not currently go to school
- A mainstream public school
- A mainstream independent / private school
- A mainstream public school with some support during the week
- A mainstream independent / private school with some support during the week
- A support class in a mainstream public school
- A support class in a mainstream independent / private school
- A specialist disability school
- Home schooled
- Other, please provide additional details: _________________________________________

1. At what age did you first notice a concern with your child’s development? Provide age in months and years.
2. From the time that you noticed a concern, how long did it take for you to be able to attend your first appointment with a health professional specialising in child development (Paediatrician, psychologist, occupational or speech therapist) for an assessment of your child’s developmental needs or diagnosis?

_______________________________________

1. From the time you noticed a concern, how long did it take for your child to receive support or intervention for your child’s needs?

- Not applicable as the assessment is being conducted
- Less than three months
- Less than six months
- Less than one year
- Less than two years
- More than two years

1. For your child attending assessment and therapy services (the child you are currently reporting on), have they been diagnosed with a developmental disorder?

- No
- Yes

If yes, what diagnoses have been made by a medical professional for your child (e.g., Autism, ADHD, Tourette syndrome)?

_______________________________________

1. Have you noticed a problem with your child’s development? This may be a problem with their social, motor or language development.

- No
- Yes

1. Does your child experience any social communication difficulties?

- No
- Yes

1. Does your child experience any language delay or problems with speech?

- No
- Yes

1. Does your child experience any restricted or repetitive behaviours?

- No
- Yes

1. How many other children do you have in your care?

- 1 child
- 2 children
- 3 children
- 4+ children

1. How many other children do you have developmental concerns about?

- 1 child
- 2 children
- 3 children
- 4+ children

***About you:***

1. How old were you (in years) when your child was born? __________________
2. What is your date of birth?

Please use format dd/mm/yyyy _ _ / _ _ / _ _ _ _

1. What is your gender?

- Male
- Female
- Other

1. What suburb do you live in? ______________
2. What is your postcode? ______________
3. Do you identify as of Aboriginal or Torres Strait Islander origin?

- No
- Yes, Aboriginal/
- Torres Strait Islander
- Yes, both

1. What country were you born in?

- Australia
- England
- China
- India
- New Zealand
- Philippines
- Vietnam
- South Africa
- Italy
- Malaysia
- Scotland
- Other (please list) _______________________________________

1. What is the main language spoken at home?

- English
- Mandarin
- Italian
- Arabic
- Cantonese
- Greek
- Vietnamese
- Other (please list) _______________________________________

1. What is your highest level of education?

- Primary School
- High School to end of year 10, 11 or 12
- TAFE or college certificate or diploma
- Trade/apprenticeship
- University degree
- Postgraduate study
- Other (please list) _______________________________________

*We will now ask some questions about your family situation. It is important that we understand your perspective.*

1. What is your relationship to this child?

- Biological mother
- Adoptive mother
- Step mother
- Foster mother
- Biological father
- Adoptive father
- Step father
- Foster father
- Grandmother
- Grandfather
- Other (please describe) _______________________________________________

1. Which situation best describes your household?

- Both biological or adoptive parents are still living in the same household
- Sole parent family after divorce/separation
- Sole parent family, other parent has never been involved or is deceased
- Step family (two parents, one being a step parent)
- Foster family
- Grandparents
- Other (please describe) ________________________________________________

1. How many other children live in the house in your care (E.g., siblings and step-siblings)

1. How many other children do you have with a diagnosed neurodevelopmental disorder? Please choose one option

- None (the child I am reporting on is the only child in my care with a diagnosed neurodevelopmental disorder)
- 1 child
- 2 children
- 3 children
- 4+ children

If your child has previously been given a diagnosis by a medical professional (e.g., Autism, ADHD, Tourette syndrome), please list them here. If not, please move on to the next question.

- - Child 1
  - Child 2 etc.

**Your experiences**

*The next few questions ask about your financial situation, including the combined income of your household and your health. We know that parenting can be both rewarding and challenging. Some of these questions may be sensitive but they help build a picture of what support and resources your family may need. (For example we know that it can sometimes be expensive and stressful.) All information provided will remain confidential and will be accessed only by authorised members of staff.*

1. What is your current work status?

- Employed full-time (35+ hours/week)
- Employed part-time or casual
- Employed, but on maternity/paternity leave
- Student, not working
- Unemployed and looking for work
- Not in paid employment, and not looking for work
- Other (describe): ________________________________________________________________

1. Before income tax is taken out, what is the current combined yearly income of everyone in your household? Include pensions and allowances.
   - No income
   - $1 - $25,999
   - $26,000 - $41,599
   - $41,600 - $64,999
   - $65,000 - $77,999
   - $78,000 - $90,999
   - $91,000 - $103,999
   - $104,000 - $155,999
   - $156,000 or more
   - Prefer not to say
2. Do you have a Medicare card?

- No
- Yes
- Prefer not to say

1. Do you have private health insurance for your child?

- No
- Yes
- Prefer not to say

1. Do you have a Health Care card?

- No
- Yes
- Prefer not to say

1. Do you have a Pensioner Concession card?

- No
- Yes
- Prefer not to say

1. Are there support agencies involved (e.g., Brighter Futures, Red Cross, Barnandos, Financial Counselling, debit assist, etc.)

- No
- Yes, please provide detail ___________________________________
- Prefer not to say

1. Are you currently a refugee or an asylum seeker?

- No
- Yes – refugee
- Yes – asylum seeker
- Prefer not to say

1. Within the past 12 months, the food you bought just didn't last and you didn't have money to get more.

- Often True
- Sometimes True
- Never True

1. Within the past 12 months, you worried whether your food would run out before you got money to buy more.

- Often True
- Sometimes True
- Never True

1. Do you have trouble paying for medicines?

- No
- Yes
- Prefer not to say

1. Do you have trouble getting transportation to medical appointments?

- No
- Yes
- Prefer not to say

1. Do you have trouble paying your heating or electricity bill?

- No
- Yes
- Prefer not to say

1. Are you currently unemployed and looking for a job?

- No
- Yes
- Prefer not to say

1. Are you interested in more education?

- No
- Yes
- Prefer not to say

**Appendix S2**

*Intervention History Questionnaire*

Please think about the intervention services or therapies that **your child** has previously received or is currently receiving when answering the following questions.

Note: do not include hours of day care when estimating how many hours of intervention have been received.

1. How many hours per week of therapy is your child currently receiving? Please choose one option.

- Services have not yet been sought
- None
- 1 - 2 hours per week
- 3 - 5 hours per week
- 6 - 10 hours per week
- 11 - 15 hours per week
- 15+ hours per week

1. Are you currently receiving any NDIS packages or services?

- No
- Yes
- Prefer not to answer

If Yes: Does NDIS cover all of your costs for support and intervention?

- No
- Yes

1. If no, how much do you estimate you will be out of pocket per year in payments for early intervention therapy? Please choose one option.

- Nothing
- Less than $1,000
- $1,000 - $5,000
- $5,001 - $10,000
- $10,001 - $20,000
- $20,001 - $30,000
- $30,001 - $40,000
- $40,001 - $50,000
- $50,000+

1. Has your child EVER received the following interventions/therapies? Select all that apply.

|  | Yes | No |
| --- | --- | --- |
| a. Paediatrician/ Child Psychiatrist |  |  |
| b. General Practitioner |  |  |
| c. Speech/Language Therapy |  |  |
| d. Occupational Therapy |  |  |
| e. Psychology/ Clinical Psychology |  |  |
| f. Physical Therapy |  |  |
| g. Behaviour Management/ Skills Therapies |  |  |
| h. NDIS service or an NDIS package |  |  |
| i. Other (please list) __________________ |  |  |

1. Is your child CURRENTLY receiving the following services/ interventions/therapies? Select all that apply.

|  | Yes | No |
| --- | --- | --- |
| a. Paediatrician/ Child Psychiatrist |  |  |
| b. General Practitioner |  |  |
| c. Speech/Language Therapy |  |  |
| d. Occupational Therapy |  |  |
| e. Psychology/ Clinical Psychology |  |  |
| f. Physical Therapy |  |  |
| g. Behaviour Management/ Skills Therapies |  |  |
| h. NDIS service or an NDIS package |  |  |
| i. Other (please list) __________________ |  |  |

1. In the past week, what is the total number of hours your child has received the relevant interventions or therapies for? Please round to the nearest half hour.

|  | Hours (list) |
| --- | --- |
| a. Paediatrician/ Child Psychiatrist |  |
| b. General Practitioner |  |
| c. Speech/Language Therapy |  |
| d. Occupational Therapy |  |
| e. Psychology/ Clinical Psychology |  |
| f. Physical Therapy |  |
| g. Behaviour Management/ Skills Therapies |  |

1. Are there any support services you think your child needs but are currently unable to access? Select all that apply.

|  | Yes |
| --- | --- |
| a. Paediatrician/ Child Psychiatrist |  |
| b. General Practitioner |  |
| c. Speech/Language Therapy |  |
| d. Occupational Therapy |  |
| e. Psychology/ Clinical Psychology |  |
| f. Physical Therapy |  |
| g. Behaviour Management/ Skills Therapies |  |
| h. NDIS service or an NDIS package |  |
| i. Other (please list) __________________ |  |

1. What have been main barriers to your ability to access these services? Select all that apply.

|  | Yes |
| --- | --- |
| a. Transport |  |
| b. Financial |  |
| c. Inability to access professionals with these skills in my region |  |
| d. Time to coordinate |  |
| e. Lack of knowledge about where to go or who to contact |  |
| f. Other (please list) __________________ |  |

***Please think about the intervention services or therapies that you, as a parent, have previously received or are currently receiving when answering the following questions.***

1. Have you EVER received the following parenting interventions/services? Select all that apply.

|  | Yes | No |
| --- | --- | --- |
| a. Positive Parenting Behaviour Support Programs |  |  |
| b. Parental Respite – In home |  |  |
| c. Parental Respite – Out of home |  |  |
| d. Parental Respite – Vacation during school camps |  |  |
| e. Parental Respite – Other (please specify) _________________ |  |  |
| f. Other (please list) __________________ |  |  |

1. Are you CURRENTLY receiving the following parenting interventions/therapies? Select all that apply.

|  | Yes | No |
| --- | --- | --- |
| a. Positive Parenting Behaviour Support Programs |  |  |
| b. Parental Respite – In home |  |  |
| c. Parental Respite – Out of home |  |  |
| d. Parental Respite – Vacation during school camps |  |  |
| e. Parental Respite – Other (please specify) _________________ |  |  |
| f. Other (please list) __________________ |  |  |

1. If it were available to you, which of the following do you think you need in the future for you and your child?

|  | Yes | No |
| --- | --- | --- |
| a. Positive Parenting Behaviour Support Programs |  |  |
| b. Parental Respite – In home |  |  |
| c. Parental Respite – Out of home |  |  |
| d. Parental Respite – Vacation during school camps |  |  |
| e. Parental Respite – Other (please specify) _________________ |  |  |
| f. Other (please list) __________________ |  |  |

**Supporting Information – References**

1. Scahill, L., et al., *Children's Yale-Brown Obsessive Compulsive Scale: reliability and validity.* J Am Acad Child Adolesc Psychiatry, 1997. **36**(6): p. 844-52.

2. Shaffer, D., et al., *A Children’s Global Assessment Scale (CGAS).* Archives of General Psychiatry, 1983. **40**: p. 1228-1231.

3. Lovibond, S.H. and P.F. Lovibond, *Manual for the Depression Anxiety Stress Scales*. 2nd ed. 1995, Sydney: Psychology Foundation.

4. Kaufman, J., et al., *Schedule for Affective Disorders and Schizophrenia for School-Age Children-Present and Lifetime Version (K-SADS-PL): Initial Reliability and Validity Data.* Journal of the American Academy of Child & Adolescent Psychiatry, 1997. **36**(7): p. 980-988.

5. Conners, C.K. and S. Goldstein, *Conners early childhood: Manual*. 2009: Multi-Health Systems Incorporated.

6. Achenbach, T.M. and L.A. Rescorla, *Manual for the ASEBA preschool forms & profiles*. 2000.

7. Achenbach, T.M. and L.A. Rescorla, *Manual for the ASEBA school-age forms & profiles.* 2001.

8. Leckman, J.F., et al., *The Yale Global Tic Severity Scale: initial testing of a clinician-rated scale of tic severity.* 1989. **28**(4): p. 566-573.

9. Swanson, J., et al., *Assessment and intervention for attention-deficit/hyperactivity disorder in the schools: Lessons from the MTA study.* 1999. **46**(5): p. 993-1009.

10. Birmaher, B., et al., *The Screen for Child Anxiety Related Emotional Disorders (SCARED): scale construction and psychometric characteristics.* J Am Acad Child Adolesc Psychiatry, 1997. **36**(4): p. 545-53.

11. Rosario, M.C., et al., *Validation of the University of São Paulo Sensory Phenomena Scale: initial psychometric properties.* CNS Spectr, 2009. **14**(6): p. 315-23.

12. Sachser, C., et al., *International development and psychometric properties of the Child and Adolescent Trauma Screen (CATS).* Journal of Affective Disorders, 2017. **210**: p. 189-195.

13. Chang, S., et al., *Initial psychometric properties of a brief parent-report instrument for assessing tic severity in children with chronic tic disorders.* 2009. **31**(3): p. 181-191.

14. Piacentini, J., et al., *BRIEF REPORT: Functional Impairment in Childhood OCD: Development and Psychometrics Properties of the Child Obsessive-Compulsive Impact Scale-Revised (COIS-R).* Journal of Clinical Child & Adolescent Psychology, 2007. **36**(4): p. 645-653.

15. Pinto, A., B. Van Noppen, and L. Calvocoressi, *Development and preliminary psychometric evaluation of a self-rated version of the Family Accommodation Scale for Obsessive-Compulsive Disorder.* Journal of obsessive-compulsive and related disorders, 2013. **2**(4): p. 457-465.

16. Foa, E.B., et al., *Development and validation of a child version of the obsessive compulsive inventory.* 2010. **41**(1): p. 121-132.

17. Gioia, G., K. Espy, and P. Isquith, *Behavior Rating Inventory of Executive Function–Preschool Version.* . 2003, Lutz, FL: PAR.

18. Gioia, G., et al., *Behavior Rating Inventory of Executive Function, Second Edition*. 2015, Lutz, FL: PAR.

19. Goodman, R., *The strengths and difficulties questionnaire: A research note. .* Journal of Child Psychology and Psychiatry and Allied Disciplines, 1997. **39**(5): p. 581-586.

20. Hidecker, M.J.C., et al., *Developing and validating the Communication Function Classification System for individuals with cerebral palsy.* 2011. **53**(8): p. 704-710.

21. Palisano, R.J., et al., *Content validity of the expanded and revised Gross Motor Function Classification System.* 2008. **50**(10): p. 744-750.

22. Eliasson, A.C., et al., *The Manual Ability Classification System (MACS) for children with cerebral palsy: scale development and evidence of validity and reliability.* Dev Med Child Neurol, 2006. **48**(7): p. 549-54.

23. Graham, H.K., et al., *The Functional Mobility Scale (FMS).* Journal of Pediatric Orthopaedics, 2004. **24**(5).

24. Jethwa, A., et al., *Development of the Hypertonia Assessment Tool (HAT): a discriminative tool for hypertonia in children.* Dev Med Child Neurol, 2010. **52**(5): p. e83-7.

25. Einspieler, C. and H.F.R. Prechtl, *Prechtl's assessment of general movements: A diagnostic tool for the functional assessment of the young nervous system.* Mental Retardation and Developmental Disabilities Research Reviews, 2005. **11**(1): p. 61-67.

26. Haataja, L., et al., *Application of a scorable neurologic examination in healthy term infants aged 3 to 8 months.* J Pediatr, 2003. **143**(4): p. 546.

27. Bayley, N., *Bayley-III: Bayley Scales of infant and toddler development*. 2009: Giunti OS.

28. Wechsler, D., *Wechsler preschool and primary scale of intelligence—fourth edition*. 2012, San Antonio, TX: The Psychological Corporation.

29. Harrison, P.L. and T. Oakland, *Adaptive Behaviour Assessment System - Third Edition Manual*. 2005, San Antonio, TX: Harcourt Assessment.

30. Wechsler, D., *Wechsler Intelligence Scale for Children - Fifth Edition*. 2014, Wechsler Intelligence Scale for Children: PsychCorp.

31. Sparrow, S., D. Cicchetti, and C. Saulnier, *Vineland Adaptive Behavior Scales, Third Edition.* 2016, Bloomington, MN: Pearson.

32. Lord, C., M. Rutter, and A. Le Couteur, *Autism Diagnostic Interview-Revised: a revised version of a diagnostic interview for caregivers of individuals with possible pervasive developmental disorders.* Journal of autism and developmental disorders, 1994. **24**(5): p. 659-685.

33. Wolraich, M.L., et al., *Psychometric properties of the Vanderbilt ADHD diagnostic parent rating scale in a referred population.* Journal of pediatric psychology, 2003. **28**(8): p. 559-568.

34. Green, E., et al., *Griffiths scales of child development*. 2016, Hogrefe Ltd.

35. Robins, D.L., et al., *The Modified Checklist for Autism in Toddlers: an initial study investigating the early detection of autism and pervasive developmental disorders.* Journal of autism and developmental disorders, 2001. **31**(2): p. 131-144.

36. Lord, C., et al., *Autism diagnostic observation schedule (ADOS): Manual*. 2008: Western Psychological Services Los Angeles.

37. Schopler, E., R.J. Reichler, and B.R. Renner, *The childhood autism rating scale (CARS)*. 2010: WPS Los Angeles.

38. Mullen, E.M., *Mullen Scales of Early Learning*. 1995, Circle Pines, MN: National Academy Press.

39. CADDRA. *CADDRA ADHD Assessment Toolkit (CAAT) forms.*; Available from: <http://www.caddra.ca/cms4/pdfs/caddraGuidelines2011_Toolkit.pdf>.

40. Roid, G.H., *Stanford-Binet Intelligence Scales, Fifth Edition*. 2003, Itasca, IL: Riverside.

41. Squires, J. and D. Bricker, *Ages & Stages Questionnaires®, Third Edition (ASQ®-3): A Parent-Completed Child Monitoring System*. 2009, Baltimore: : Paul H. Brookes Publishing Co., Inc.

42. Wechsler, D., *Wechsler Individual Achievement Test, Third Edition*. 2009, San Antonio, TX: Psychological Corporation.

43. Spence, S.H., *A measure of anxiety symptoms among children.* Behaviour research and therapy, 1998. **36**(5): p. 545-566.

44. Manly, T., et al., *Tests of Everyday Attention for Children, Second Edition (TEA-Ch2)*. 2016, London, UK: Pearson Education Ltd.

45. Lam, K.S.L. and M.G. Aman, *The Repetitive Behavior Scale-Revised: Independent Validation in Individuals with Autism Spectrum Disorders.* Journal of Autism and Developmental Disorders, 2007. **37**(5): p. 855-866.

46. Korkman, M., U. Kirk, and S. Kemp, *NEPSY II: Clinical and interpretive manual*. 2007: Harcourt Assessment, PsychCorp.

47. Aman, M.G., et al., *The aberrant behavior checklist: a behavior rating scale for the assessment of treatment effects.* American journal of mental deficiency, 1985.

48. Semel, E., E.H. Wiig, and W. Secord, *Clinical Evaluation of Laguage Fundamentals - Fifth Edition*. 2013, San Antonio, TX.: Pearson.

49. Einfeld, S.L. and B.J. Tonge, *The Developmental Behavior Checklist: The development and validation of an instrument to assess behavioral and emotional disturbance in children and adolescents with mental retardation.* Journal of autism and developmental disorders, 1995. **25**(2): p. 81-104.

50. Bishop, D., *The Children's communication checklist: CCC-2 manual*. 2012: Pearson.

51. Berument, S.K., et al., *Autism screening questionnaire: diagnostic validity.* The British Journal of Psychiatry, 1999. **175**(5): p. 444-451.

52. Constantino, J.N. and C.P. Gruber, *Social responsiveness scale second edition (SRS-2): Manual*. 2012: Western Psychological Services (WPS).

53. McIntosh, D.N., et al., *Overview of the short sensory profile.*, in *Sensory profile user’s manual.*, W. Dunn, Editor. 1999, Psychological Corporation San Antonio, TX. p. 59-73.

54. Fairburn, C.G., Z. Cooper, and M. O'Connor, *The eating disorder examination.* International Journal of Eating Disorders, 1993. **6**: p. 1-8.

55. Fairburn, C.G. and S.J. Beglin, *Assessment of eating disorders: Interview or self‐report questionnaire?* International journal of eating disorders, 1994. **16**(4): p. 363-370.

56. Chorpita, B.F., et al., *Assessment of symptoms of DSM-IV anxiety and depression in children: A revised child anxiety and depression scale.* Behaviour research and therapy, 2000. **38**(8): p. 835-855.

57. Baron-Cohen, S., et al., *The autism-spectrum quotient (AQ): Evidence from asperger syndrome/high-functioning autism, malesand females, scientists and mathematicians.* Journal of autism and developmental disorders, 2001. **31**(1): p. 5-17.

58. Folio, M.R. and R.R. Fewell, *Peabody Developmental Motor Scales Examiner's Manual*. 2nd ed. 2000, Austin, TX: Pro-Ed.

59. Skuse, D., et al., *The developmental, dimensional and diagnostic interview (3di): a novel computerized assessment for autism spectrum disorders.* Journal of the American Academy of Child & Adolescent Psychiatry, 2004. **43**(5): p. 548-558.

60. Brannan, A.M., C.A. Heflinger, and L. Bickman, *The Caregiver Strain Questionnaire: Measuring the Impact on the Family of Living with a Child with Serious Emotional Disturbance.* Journal of Emotional and Behavioral Disorders, 1997. **5**(4): p. 212-222.

61. Henderson, S.E., D.A. Sugden, and A.L. Barnett, *Movement assessment battery for children*. Vol. 26. 2007: Harcourt Assessment London.

62. Goodman, R., et al., *The development and well‐being assessment: Description and initial validation of an integrated assessment of child and adolescent psychopathology.* Journal of child psychology and psychiatry, 2000. **41**(5): p. 645-655.

63. Üstün, T.B., et al., *Measuring health and disability: Manual for WHO disability assessment schedule WHODAS 2.0*. 2010: World Health Organization.

64. Hamilton, M., *A rating scale for depression.* J Neurol Neurosurg Psychiatry, 1960. **23**(1): p. 56-62.

65. Wetherby, A.M. and B.M. Prizant, *Communication and symbolic behavior scales: Developmental profile*. 2002: Paul H Brookes Publishing Co.

66. Riley, A.W., et al., *The Parent Report Form of the CHIP-Child Edition: reliability and validity.* Med Care, 2004. **42**(3): p. 210-20.

67. Conners, C., D. Erhardt, and E. Sparrow, *The Conners adult ADHD rating scale (CAARS)*. 1998, Toronto: Multi-Health Systems Inc.,.

68. Dale, P.S., *The validity of a parent report measure of vocabulary and syntax at 24 months.* Journal of Speech, Language, and Hearing Research, 1991. **34**(3): p. 565-571.

69. Cohen, I.L., et al., *The PDD Behavior Inventory: a rating scale for assessing response to intervention in children with pervasive developmental disorder.* Journal of autism and developmental disorders, 2003. **33**(1): p. 31-45.

70. Kessler, R.C., et al., *Short screening scales to monitor population prevalences and trends in non-specific psychological distress.* Psychological medicine, 2002. **32**(6): p. 959.

71. Gowers, S.G., et al., *Brief scale for measuring the outcomes of emotional and behavioural disorders in children.* The British Journal of Psychiatry, 1999. **174**(5): p. 413-416.

72. Allison, C., et al., *The Q-CHAT (Quantitative CHecklist for Autism in Toddlers): a normally distributed quantitative measure of autistic traits at 18-24 months of age: preliminary report.* J Autism Dev Disord, 2008. **38**(8): p. 1414-25.

73. Swanson, J., et al., *Over-identification of extreme behavior in the evaluation and diagnosis of ADHD/HKD.* Accessible online at http:\\www. ADHD. net Accessed February, 2001. **20**.

74. Achenbach, T.M. and L.A. Rescorla, *Manual for the ASEBA adult forms & profiles*. 2003, Burlington, VT, USA: Research Center for Children, Youth, & Families, University of Vermont.

75. Sheslow, D. and W. Adams, *Wide Range Assessment of Memory and Learning, Second Edition*. 2003, Lutz, FL: Psychological Assessment Resources.

76. Adams, W. and D. Sheslow, *Wide Range Assessment of Visual Motor Abilities*. 1995, Wilmington, DE: Wide Range.

77. Engel Jr, J.V.N.P., *Outcome with respect to epileptic seizures.* Surgical treatment of the epilepsies, 1993: p. 609-621.

78. Statistics, A.B.o., *Technical Paper: Socio-economic Indexes for Areas (SEIFA).* . 2016: Canberra, Australia.

79. Msall, M.E., et al., *The Functional Independence Measure for Children (WeeFIM) conceptual basis and pilot use in children with developmental disabilities.* Clinical pediatrics, 1994. **33**(7): p. 421-430.

80. Linacre, J.M., et al., *The structure and stability of the functional independence measure.* Archives of Physical Medicine and Rehabilitation, 1994. **75**(2): p. 127-132.

81. Varni, J.W., M. Seid, and P.S. Kurtin, *PedsQL 4.0: reliability and validity of the Pediatric Quality of Life Inventory version 4.0 generic core scales in healthy and patient populations.* Med Care, 2001. **39**(8): p. 800-812.

82. Zimmerman, I.L., V.G. Steiner, and R.E. Pond, *Preschool language scales, fifth edition – Australian and New Zealand language adapted edition (PLS-5).* 2012, Camberwell VIC, Australia: Pearson Australia Group.

83. Hoffman, L., et al., *Assessing family outcomes: Psychometric evaluation of the beach center family quality of life scale.* Journal of marriage and family, 2006. **68**(4): p. 1069-1083.

84. WHO, *International classifcation of functioning, disability and health: ICF*. 2001: World Health Organization, Geneva.

85. Ravens-Sieberer, U., et al., *Feasibility, reliability, and validity of the EQ-5D-Y: results from a multinational study.* Quality of Life Research, 2010. **19**(6): p. 887-897.

86. Lamsal, R., et al., *Generic preference-based health-related quality of life in children with neurodevelopmental disorders: a scoping review.* Dev Med Child Neurol, 2020. **62**(2): p. 169-177.

87. Peasgood, T., et al., *The impact of ADHD on the health and well-being of ADHD children and their siblings.* Eur Child Adolesc Psychiatry, 2016. **25**(11): p. 1217-1231.

88. Sousa, M.Á.P., P.R.O. Sánchez-Toledo, and N.G.J.A.A.P. Fuertea, *Parent-child discrepancy in the assessment of health-related quality of life using the EQ-5D-Y questionnaire.* 2017. **115**(6): p. 541-546.

89. Rowen, D., et al., *A Review of the Psychometric Performance of Selected Child and Adolescent Preference-Based Measures Used to Produce Utilities for Child and Adolescent Health.* Value Health, 2021. **24**(3): p. 443-460.

90. Janssens, A., et al., *Measurement properties of multidimensional patient-reported outcome measures in neurodisability: a systematic review of evaluation studies.* Dev Med Child Neurol, 2016. **58**(5): p. 437-51.

91. Stevens, K., *Valuation of the Child Health Utility 9D Index.* PharmacoEconomics, 2012. **30**(8): p. 729-747.

92. Furber, G. and L. Segal, *The validity of the Child Health Utility instrument (CHU9D) as a routine outcome measure for use in child and adolescent mental health services.* Health Qual Life Outcomes, 2015. **13**: p. 22.

93. Wolf, R.T., et al., *The longitudinal validity of proxy-reported CHU9D.* Quality of Life Research, 2021. **30**(6): p. 1747-1756.

94. Limbers, C.A., et al., *Patient-reported Pediatric Quality of Life Inventory™ 4.0 Generic Core Scales in pediatric patients with attention-deficit/hyperactivity disorder and comorbid psychiatric disorders: feasibility, reliability, and validity.* Value Health, 2011. **14**(4): p. 521-30.

95. Hiscock, H., et al., *Impact of a behavioural sleep intervention on symptoms and sleep in children with attention deficit hyperactivity disorder, and parental mental health: randomised controlled trial.* BMJ : British Medical Journal, 2015. **350**: p. h68.

96. Tavernor, L., et al., *Finding out what matters: validity of quality of life measurement in young people with ASD.* Child: Care, Health & Development, 2013. **39**(4): p. 592-601.

97. O’Hare, D., et al., *Factors impacting the quality of peer relationships of youth with Tourette’s syndrome.* 2015. **3**(1): p. 1-13.

98. Varni, J.W., et al., *The PedsQL™* 4.0 as a Pediatric Population Health Measure: Feasibility, Reliability, and Validity.* Ambulatory Pediatrics, 2003. **3**(6): p. 329-341.

99. Viecili, M.A. and J.A. Weiss, *Reliability and Validity of the Pediatric Quality of Life Inventory With Individuals With Intellectual and Developmental Disabilities: AJMR.* American Journal on Intellectual and Developmental Disabilities, 2015. **120**(4): p. 289-301,371,373.

100. Lamsal, R., D.J. Dutton, and J.D. Zwicker, *Using the ages and stages questionnaire in the general population as a measure for identifying children not at risk of a neurodevelopmental disorder.* BMC Pediatr, 2018. **18**(1): p. 122.

101. Russell, M.J., et al., *Health care service for families with children at early risk of developmental delay: an All Our Families cohort study.* Developmental Medicine & Child Neurology, 2020. **62**(3): p. 338-345.

102. Hardy, S., et al., *Can Screening with the Ages and Stages Questionnaire Detect Autism?* Journal of developmental and behavioral pediatrics : JDBP, 2015. **36**(7): p. 536-543.

103. Hornman, J., et al., *Validity and internal consistency of the Ages and Stages Questionnaire 60-month version and the effect of three scoring methods.* Early Hum Dev, 2013. **89**(12): p. 1011-5.

104. Schonhaut, L., et al., *Validity of the Ages and Stages Questionnaires in Term and Preterm Infants.* Pediatrics, 2013. **131**(5): p. e1468.

105. Iizuka, C., et al., *Comparison of the strengths and difficulties questionnaire (SDQ) scores between children with high-functioning autism spectrum disorder (HFASD) and attention-deficit/hyperactivity disorder (AD/HD).* Brain and Development, 2010. **32**(8): p. 609-612.

106. McMahon, J., et al., *Anxiety in children and adolescents with cerebral palsy.* Journal of Paediatrics and Child Health, 2020. **56**(8): p. 1194-1200.

107. Bourdon, K.H., et al., *The Strengths and Difficulties Questionnaire: U.S. Normative Data and Psychometric Properties.* Journal of the American Academy of Child & Adolescent Psychiatry, 2005. **44**(6): p. 557-564.

108. Stone, L.L., et al., *Psychometric properties of the parent and teacher versions of the strengths and difficulties questionnaire for 4- to 12-year-olds: a review.* Clinical child and family psychology review, 2010. **13**(3): p. 254-274.

109. Hawes, D.J. and M.R. Dadds, *Australian data and psychometric properties of the Strengths and Difficulties Questionnaire.* Australian & New Zealand Journal of Psychiatry, 2004. **38**(8): p. 644-651.

110. Bergstrom, M. and S. Baviskar, *A Systematic Review of Some Reliability and Validity Issues regarding the Strengths and Difficulties Questionnaire Focusing on Its Use in Out-of-Home Care.* J Evid Based Soc Work (2019), 2021. **18**(1): p. 1-31.

111. Zlomke, K.R., *Psychometric properties of internet administered versions of Penn State Worry Questionnaire (PSWQ) and Depression, Anxiety, and Stress Scale (DASS).* Computers in Human Behavior, 2009. **25**(4): p. 841-843.

112. Norton, P., *Depression Anxiety and Stress Scales (DASS-21): Psychometric analysis across four racial groups.* Anxiety, Stress & Coping, 2007. **20**(3): p. 253-265.

113. Henry, J.D. and J.R. Crawford, *The short-form version of the Depression Anxiety Stress Scales (DASS-21): Construct validity and normative data in a large non-clinical sample.* British Journal of Clinical Psychology, 2005. **44**(2): p. 227-239.

114. Sinclair, S.J., et al., *Psychometric Evaluation and Normative Data for the Depression, Anxiety, and Stress Scales-21 (DASS-21) in a Nonclinical Sample of U.S. Adults.* Evaluation & the Health Professions, 2011. **35**(3): p. 259-279.

115. Ustun, B., et al., *The World Health Organization Adult Attention-Deficit/Hyperactivity Disorder Self-Report Screening Scale for DSM-5.* JAMA Psychiatry, 2017. **74**(5): p. 520-526.

116. Kessler, R.C., et al., *Validity of the World Health Organization Adult ADHD Self-Report Scale (ASRS) Screener in a representative sample of health plan members.* Int J Methods Psychiatr Res, 2007. **16**(2): p. 52-65.

117. Brevik, E.J., et al., *Validity and accuracy of the Adult Attention-Deficit/Hyperactivity Disorder (ADHD) Self-Report Scale (ASRS) and the Wender Utah Rating Scale (WURS) symptom checklists in discriminating between adults with and without ADHD.* Brain Behav, 2020. **10**(6): p. e01605.

118. Brannan, A.M., M.M. Athay, and A.R.V. de Andrade, *Measurement quality of the caregiver strain questionnaire-short form 7 (CGSQ-SF7).* Adm Policy Ment Health, 2012. **39**(51).

119. Hodge, M.A., et al., *Predictors of adaptive functioning in preschool aged children with autism spectrum disorder.* Autism Research, 2021. **n/a**(n/a).

120. Craig, F., et al., *Overlap Between Autism Spectrum Disorders and Attention Deficit Hyperactivity Disorder: Searching for Distinctive/Common Clinical Features.* Autism Research, 2015. **8**(3): p. 328-337.

121. Voorman, J.M., et al., *Social functioning and communication in children with cerebral palsy: association with disease characteristics and personal and environmental factors.* Developmental Medicine and Child Neurology, 2010. **52**(5): p. 441-7.

122. Hill, T.L., et al., *Vineland III*, in *Encyclopedia of Autism Spectrum Disorders*, F.R. Volkmar, Editor. 2017, Springer New York: New York, NY. p. 1-4.

123. Strang, J.F., et al., *Increased Gender Variance in Autism Spectrum Disorders and Attention Deficit Hyperactivity Disorder.* Archives of Sexual Behavior, 2014. **43**(8): p. 1525-1533.

124. Lawson, R., et al., *Everyday Executive Function Impairments Predict Comorbid Psychopathology in Autism Spectrum and Attention Deficit Hyperactivity Disorders. .* Neuropsychology, 2015. **29**(3): p. 445-453.

125. Sigurdardottir, S., et al., *Behavioural and emotional symptoms of preschool children with cerebral palsy: a population-based study.* Developmental Medicine and Child Neurology, 2010. **52**(11): p. 1056-1061.

126. Nakamura, B.J., et al., *A Psychometric Analysis of the Child Behavior Checklist DSM-Oriented Scales.* Journal of Psychopathology and Behavioral Assessment, 2009. **31**(3): p. 178-189.

127. Pandolfi, V., C.I. Magyar, and C.A. Dill, *An initial psychometric evaluation of the CBCL 6–18 in a sample of youth with autism spectrum disorders.* Research in Autism Spectrum Disorders, 2012. **6**(1): p. 96-108.

128. DeLuca, H., A. Sorgente, and M.H. van Dulmen, *Dyadic Invariance of the Adult Self-Report and Adult Behavior Checklist.* Psychological Assessment, 2019. **31**(2): p. 192-209.

129. Mahone, E.M., et al., *Validity of the behavior rating inventory of executive function in children with ADHD and/or Tourette syndrome.* Arch Clin Neuropsychol, 2002. **17**(7): p. 643-62.

130. Gioia, G.A., et al., *Confirmatory factor analysis of the Behavior Rating Inventory of Executive Function (BRIEF) in a clinical sample.* Child neuropsychology : a journal on normal and abnormal development in childhood and adolescence, 2002. **8**(4): p. 249-257.

131. Hovik, K.T., et al., *Distinct Patterns of Everyday Executive Function Problems Distinguish Children With Tourette Syndrome From Children With ADHD or Autism Spectrum Disorders.* Journal of Attention Disorders, 2014. **21**(10): p. 811-823.

132. Whittingham, K., et al., *Everyday psychological functioning in children with unilateral cerebral palsy: does executive functioning play a role?* Developmental Medicine & Child Neurology, 2014. **56**(6): p. 572-579.

133. Roth, R.M., P.K. Isquith, and G.A. Gioia, *Assessment of Executive Functioning Using the Behavior Rating Inventory of Executive Function (BRIEF)*, in *Handbook of Executive Functioning*, S. Goldstein and J.A. Naglieri, Editors. 2014, Springer New York: New York, NY. p. 301-331.

134. Bausela Herreras, E., *BRIEF-P: Validation Study in Children in Early Childhood With Neurodevelopmental Disorders.* SAGE Open, 2019. **9**(3): p. 2158244019879166.

135. McCarthy, M.L., et al., *The Pediatric Quality of Life Inventory: An Evaluation of Its Reliability and Validity for Children With Traumatic Brain Injury.* Archives of Physical Medicine and Rehabilitation, 2005. **86**(10): p. 1901-1909.

136. Reddy, L.A., J.B. Hale, and L.K. Brodzinsky, *Discriminant validity of the Behavior Rating Inventory of Executive Function Parent Form for children with attention-deficit/hyperactivity disorder.* School Psychology Quarterly, 2011. **26**(1): p. 45-55.
